# Diagnostic delay, care needs, and trust in healthcare among persons with rare disease and their next of kin living in Sweden

**DOI:** 10.1007/s12687-026-00931-6

**Published:** 2026-08-20

**Authors:** Maria Johansson Soller, Jamie Linnea Luckhaus, Stephanie Juran, Charlotta Ingvoldstad Malmgren

**Affiliations:** 1 Department of Molecular Medicine and Surgery, Karolinska Institutet, Stockholm, Sweden; 2 Center for Research Ethics and Bioethics, Uppsala University, Uppsala, Sweden; 3 Department of Women’s and Children’s Health, Participatory eHealth and Health Data, Uppsala University, Uppsala, Sweden; 4 Rare Diseases Sweden, Swedish Umbrella Organization for Patients and Relatives, Sundbyberg, Sweden; 5 Department of Clinical Genetics, Akademiska University Hospital, Uppsala, Sweden; 6 Center for Rare Diseases, Clinical Genetics, Karolinska University Hospital, Stockholm, Sweden

**Keywords:** Rare disease, Diagnostic journey, Health care needs, Healthcare access, Healthcare satisfaction, Trust in healthcare

## Abstract

In Sweden, it is estimated that around half a million people live with a rare disease, and many more are affected through a family member. Despite this, little is known about patients’ diagnostic journey, healthcare needs, and trust in the healthcare system. The study provides valuable insight into the diagnostic journey and healthcare experiences of individuals with rare diseases and their next of kin. An online cross-sectional survey was completed in 2021 by members of Rare Diseases Sweden. In total, 942 respondents representing 120 different diagnoses participated. Over half were people living with a rare disease (PLWRD), while the remaining respondents were caregivers to PWLRD (P-PWLRD). Slightly more than one third of the conditions involve intellectual disability, and one in five include motor impairment. Almost half involve complex healthcare needs. Most respondents were diagnosed as adults (44.4%), followed by childhood (27.9%) and at birth (16.7%). More than one third were diagnosed within six months of first healthcare contact, and nearly half within one year, while one in ten waited over ten years. Seven out of ten have consulted an expert in their condition. Those with diagnostic delays exceeding ten years were less likely to see a specialist, more likely to perceive unmet healthcare needs, and reported lower trust in healthcare and healthcare professionals.

**Supplementary Information:**

The online version contains supplementary material available at https://doi.org/10.1007/s12687-026-00931-6.

## Introduction

In Europe, including Sweden, a rare disease (RD) is defined as a disease affecting fewer than 1 in 2000 individuals in a population (EURORDIS-Rare Diseases Europe), but collectively as many as 3.5–5.9% of the population are affected, due to the large number of different RDs. It is estimated that approximately 500 000 individuals in Sweden, and hundreds of millions worldwide are affected by RD (Wang et al 2024). There are currently at least 6 000–8 000 different identified RDs (Ferreira 2019; Smith, et al. 2022), of which up to 80% are believed to have a genetic origin (Nguengang Wakap et al. 2020; Marwaha et al. 2022). Phenotypes can range from mild symptoms in a single organ, for example eyes, to syndromes affecting multiple organs, sometimes with severe neurological symptoms and intellectual disabilities. Many RDs manifest in childhood, but some later in life; they are often life-long, impact quality of life, and may lead to a shorter life span (Nguengang Wakap et al. 2020). Living with an RD or having a child with a rare condition not only affects the physical health of the affected individual but also often comes with psychosocial or even mental health issues due to lifelong circumstances (www.rareminds.org). The affected individual, as well as their parents, often need to have the role of an expert, coordinator, and advocate (Bauskis et al 2022).

A genetic diagnosis can help in understanding the underlying cause, assessing prognosis, planning surveillance and estimating familial risk. Although modern sequencing technologies have reduced the average time between disease onset and a genetic diagnosis here referred to as the diagnostic journey—this interval can still be lengthy (Bauskis et al. 2022; Tesi et al. 2023; Reed et al. 2024). Delays may occur due to factors such as not being referred for genetic testing, inappropriate testing, or because testing was performed at a time that current available testing lacked the method itself or sensitivity or resolution needed. Diagnostic yields for rare diseases range from 20 to 50%, depending on inclusion criteria (defined symptoms) for the referral for genetic testing, where severe multiorgan syndromes show a higher diagnostic rate (Wright et al. 2023). Even with modern comprehensive approaches such as whole-genome sequencing (WGS), more than 60% of cases might remain genetically undiagnosed (reviewed in Tesi et al. 2023). Therefore, many patients only have a clinical diagnosis based purely on their symptoms.

The Swedish healthcare system is primarily tax-funded, and composed of about 44 000 physicians, including around 7 000 general practitioners (GPs) that work in primary care, but only approximately 100 clinical geneticists. General medicine is responsible for managing a wide range of cases in Sweden, including infections, minor injuries, asthma, type 2 diabetes, hypertension, heart failure, depression, chronic pain, maternity care, and pediatric care. The healthcare system is structured so that a patient’s first point of contact is typically their GP, who then refers them to a specialist as necessary (1177.se, Vårdgarantin 2024). Many individuals with RDs report challenges in access to a GP, as their disease is often considered too complicated for primary care, even when they might only be facing common infections or symptoms unrelated to their rare condition, which are otherwise generally treated by a GP (1177.se, Vårdgarantin 2024). Also, many of them have experienced a lack of referrals to specialist care, and many times they themselves need to coordinate their healthcare contacts, (www.sallsyntadiagnoser.se; Briscoe et al. 2025; Hemmesch, et al. 2025). Often, there is also a lack of experts on the specific rare condition (expert is here defined as a HC professional with satisfactory knowledge about the RDs, their treatment and follow-up) (Hemmesch et al. 2025).

Furthermore, Swedish healthcare is divided into 7 larger independently run regions. This organization of healthcare results in care inequalities and injustice for patients both related to genetic diagnosis and care, even if “equal care” is a fundamental principle in the Swedish Health and Medical Services Act (HSL; SFS 2017:30). Differences in care access between regions remains a great challenge, including genomic testing, specialty care and specific experts, impacting RD diagnosis and care.

Trust and confidence in healthcare professionals are likely to be influenced by factors mentioned above, but how common these experiences are and how they influence their experiences of healthcare is not well studied (Jones et al. 2024).

Our study aims to deepen the knowledge about possible diagnostic delays for PLWRD and how it might affect their access to different healthcare sectors. We also aim to explore the trust in healthcare and healthcare professionals among people living with a rare disease (PLWRD) and their caregivers or parents (P-PLWRD), as well as their care needs met.

## Methods

The study is reported in accordance with the STROBE checklist for cross-sectional studies (see Appendix 1).

### Study design and setting

**Fig. 1:**
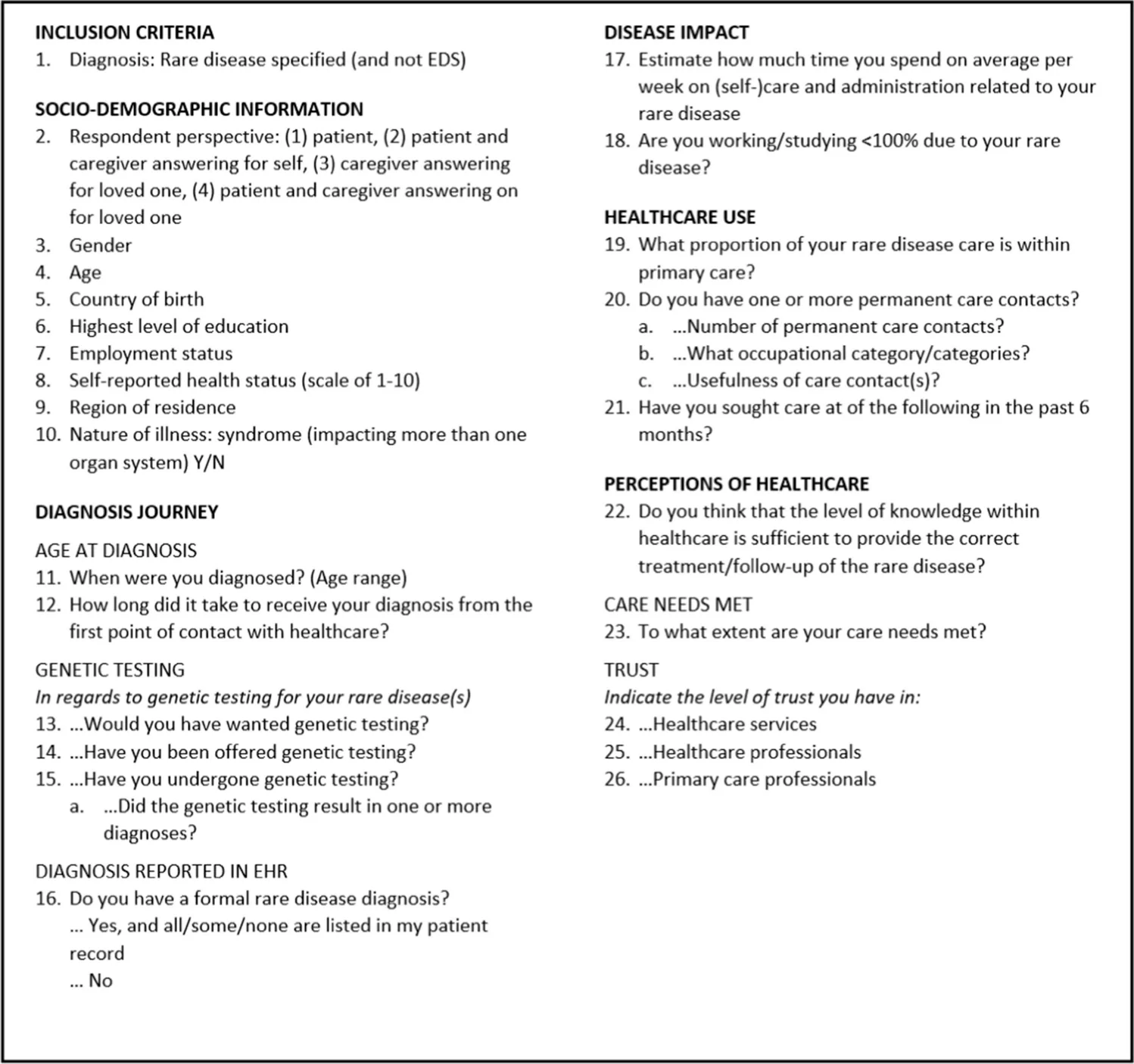
Survey questions included in the analysis

We analyzed the responses to an online survey carried out by *Rare Diseases Sweden* (sallsyntadiagnoser.se), which consisted of 105 items: single-choice, multiple-choice, likert scale, and free-text items (see Fig. 1). Survey sections covered *socio-demographic information*, *diagnosis journey* including genetic testing and time to diagnosis, *disease impact* on daily life, *healthcare use* and *perceptions of healthcare* quality. The estimated time to fill in the survey was 60 min, depending on how many free-text questions participants chose to answer. None of the items were mandatory, and some questions appeared conditionally based on the respondent’s previous response.

The survey was designed in Swedish by *Rare Diseases Sweden* in collaboration with the Center for Rare Diagnoses at Karolinska University hospital, LIF—The Research-based Pharmaceutical Industry in Sweden, the Swedish Agency for Health Technology and Assessment of Social Services (SBU), the Dental and Pharmaceutical benefits agency (TLV), and Sweden’s three specialized dental care centers of rare conditions, the National Board of Health and Welfare (Socialstyrelsen), and the Swedish Agency for Health and Care Services (Vårdanalys). Drafting of survey sections and questions was based on the over 25 years of experience from RD Sweden, including over 20 years of disseminating member surveys. The survey development was led by an experienced researcher (SJ) and some questions were adapted to match large Swedish healthcare surveys. No formal validation was performed because data was initially collected as a member survey. After finishing, the dataset was approved as a register by the Swedish ethical review authority, opening up for scientific analysis and publication. The original survey can be found in Appendix 2.

The survey was open for one month (November 29th, 2021—December 22nd, 2021) and shared with the 8,594 members of *Rare Diseases Sweden* who had provided contact information. PLWRD and P-PLWRD —answering on behalf of the family member with a rare disease (most commonly their child), were invited to complete the survey. Respondents under 16 were required to complete the survey together with an adult. The survey was distributed digitally using a commercial survey company (Stoswe.se), who sent out a unique survey link to each participant via e-mail or SMS, depending on contact details provided. Participants who did not complete the study received up to two reminders. This resulted in 1 481 responses, which gave a response rate of 17.3%. Since the survey comprises over 100 questions and an average time for answering was about 60 min, the response rate was evaluated as satisfactory. There are currently no other data sources addressing life and healthcare situations for PLWRD in Sweden. Confirmation of the reported RDs was not possible, as responses were anonymous.

### Inclusion criteria

Respondents could select from 128 different RDs, write in (free-text), or choose "prefer not to specify.” To achieve our sample, we excluded anyone who did not specify which RD diagnosis (*n* = 231) because we wanted to deepen the analysis by taking the nature of the diagnoses into account. We also excluded individuals with Ehlers-Danlos Syndrome (*n* = 310), as they were the focus of a separate paper (Luckhaus et al. 2025). Any written-in responses of the diagnoses were manually sorted by a clinician (MJS). This resulted in a total sample of 942 patients (see Tables 1, 2).

**Table 1:** Sample compared to initial survey sample

	Total sample
Original sample size	1 483
Specified RD	
Yes, reported Ehlers-Danlos Syndrome (EDS/HDS)	−310
No, did not specify	−231
Yes, reported diagnosis other than EDS/HDS	**942** (our sample)

For exact wording of the survey items, refer to Appendix 2 and 3

**Table 2:** Usual age of disease onset compared to actual age diagnosed and time to diagnosis

The usual age of onset for the reported diagnosis (*n* = 935)	Congenital/ Newborn *n* = 216 (%)	Toddler *n* = 122 (%)	Adulthood, *n* = 37 (%)	Varies, *n* = 562 (%)
Actual age at respondent’s diagnosis				
During pregnancy	3.3	-	-	1.1
At birth	46.3	12.4	-	-
Childhood	34.1	65.3	-	-
Teenage	2.3	3.3	-	-
Adulthood	4.7	4.7	86.5	62.9
Time to receive a diagnosis				
< 6 months	49.5	33.6	27	33
7–11 months	6.5	9	18.9	11.9
1–2 years	5.6	17.2	5.4	11.9
3–4 years	9.8	8.2	10.8	7.3
4–10 years	7.9	13.9	10.8	8.7
> 10 years	6.1	5.7	8.1	13.9
*Other* or* don’t know*	14.5	12.3	18.2	13.2

#### The excluded respondent subgroup

Those who did not state their diagnosis in the survey (*n* = 230) were excluded from the analysis, since we could not classify them (e.g. motor impairment or not) based on their disease. Of these excluded respondents, nearly half (47.6%, 108/227) were unsure as to whether they have a clinical diagnosis and whether it is reported in their EHR, and nearly a third (33.9%, 77/227) responded that they do not have a clinical diagnosis. Out of the 230 who did not specify their diagnosis, 34 individuals (34/230, 14.8%) reported that they were diagnosed via genetic testing without stating the gene. Note that all survey respondents were members of *Rare Diseases Sweden*.

### Analysis

We used 26 questions from *Rare Diseases Sweden*’s member survey from 2021 (see Fig. 1). Analysis consisted of descriptives (count, percentage) summarized through frequency tables and in-text descriptions, and statistical association testing, described in more detail below. On survey items that had missing responses, calculations were performed on available data only. Where “I don’t know” or “Not relevant” were possible answer options, these were not treated as missing data but were instead reported. Likert items were dichotomized where applicable i.e. combining “high” and “very high”.

A Clinical Geneticist (MJS) labelled respondents according to if the diagnosis provided as described having the following yes/no/in some cases: motor impairment, intellectual disability, a complex syndrome (defined as a condition impacting more than three organ systems), and a known genetic cause using several resources like Orphanet.org, OMIM.org and pubmed.ncbi.nlm.nih.gov (Date 241101). Respondents were also labelled according to the usual age of onset for their specific diagnosis, as to be able to assess the time between onset and the genetic diagnosis. The clinician defined variables *motor impairment*, *intellectual disability*, and *complex syndrome* according with symptoms might be present in each specific RD, as well as the respondent-reported variables *proportion of care within primary care* and *length of diagnosis journey* were pre-selected by all authors as subgrouping variables to form a basis of the comparative analysis. Associations between these participant subgroup characteristics and four outcome variables — trust in healthcare providers (HCPs), trust in primary care (PC), trust in the healthcare system (HC), and perceived extent to which care needs were met — were examined using chi-square tests of independence by JLL. For each subgrouping variable × outcome combination, contingency tables were produced and Pearson’s chi-square statistic (χ^2^), degrees of freedom (df), sample size (N), and effect size (Cramér’s V) were reported. Effect sizes were interpreted according to Cohen’s guidelines (small ≈ 0.10, medium ≈ 0.30, large ≈ 0.50).

To explore the nature of significant associations, standardized residuals were examined for each cell. Standardized residuals ≥|2| were interpreted as indicating a meaningful deviation from the expected frequency under the null hypothesis, with positive values representing overrepresentation and negative values representing underrepresentation. Given the large number of comparisons (8 subgrouping variables × 4 outcome variables), *p*-values from the chi-square tests were adjusted for multiple testing using the Benjamini–Hochberg false discovery rate (FDR) procedure. Both unadjusted and FDR-adjusted *p*-values are reported, with statistical significance set at *α* = 0.05 (two-tailed). Analyses were conducted in JASP (version 0.19.1) under the Contingency Tables module.

## Compliance with ethics guidelines

Scientific analysis of survey results received ethical approval from the Ethical Review Authority in Sweden (Approval #2023–06900-01–435533) (Swedish Ethics review Act). The survey responses were anonymous.

All procedures followed were in accordance with the ethical standards of the responsible committee for human experimentation (institutional and national) and with the Helsinki Declaration of 1975, as revised in 2000. Informed consent was obtained from all patients responding to the survey.

## Results

Survey results were translated and described below. As the questions were skippable, the number of respondents varied from question to question, and the respondents (*n*) are presented in a table or else in text (xx/xx).

### Characteristics, *n* = 942

Among the 942 respondents, 56% (528/940) reported living with a rare disease (RD), and 55% (517/940) completed the survey from their own perspective (PLWRD). Meanwhile, 44.4% (417/940) were responding on behalf of a person living with an RD (P-PLWRD). These subgroups were not mutually exclusive: 12.4% (116/940) of respondents both lived with an RD and were also next of kin to another PLWRD (See Supplementary Table 1).

A surplus of respondents were women (58.8%, 554/940). The vast majority (96.1%, 899/940) were born in Sweden and a third were 55 or older (313/940, 33.3%). Over a third (38.64%, 301/779) were employed; nearly a third(29%, 226/779) were in school, and 14.9% (116/779) were unemployed.Geographical distribution corresponded with the country’s population density and with the distribution of members in *Rare Diseases Sweden*, with nearly a quarter in Stockholm region (24.8%, 234/941) followed by 16.5% in Western Götaland (155/941), and 10.4% in Scania (Skåne) (98/941).

Over a third (35.7%, 298/834) rated their health as 4 or below on a scale of 1–10 with lower being poorer, while the majority rated it as 5 or above (64.3%, 536/834). As for the extremes, 2.8% (23/834) reported their health as “poor” and 8.4% (70/834) rated their health as “excellent.”

**Fig. 2:**
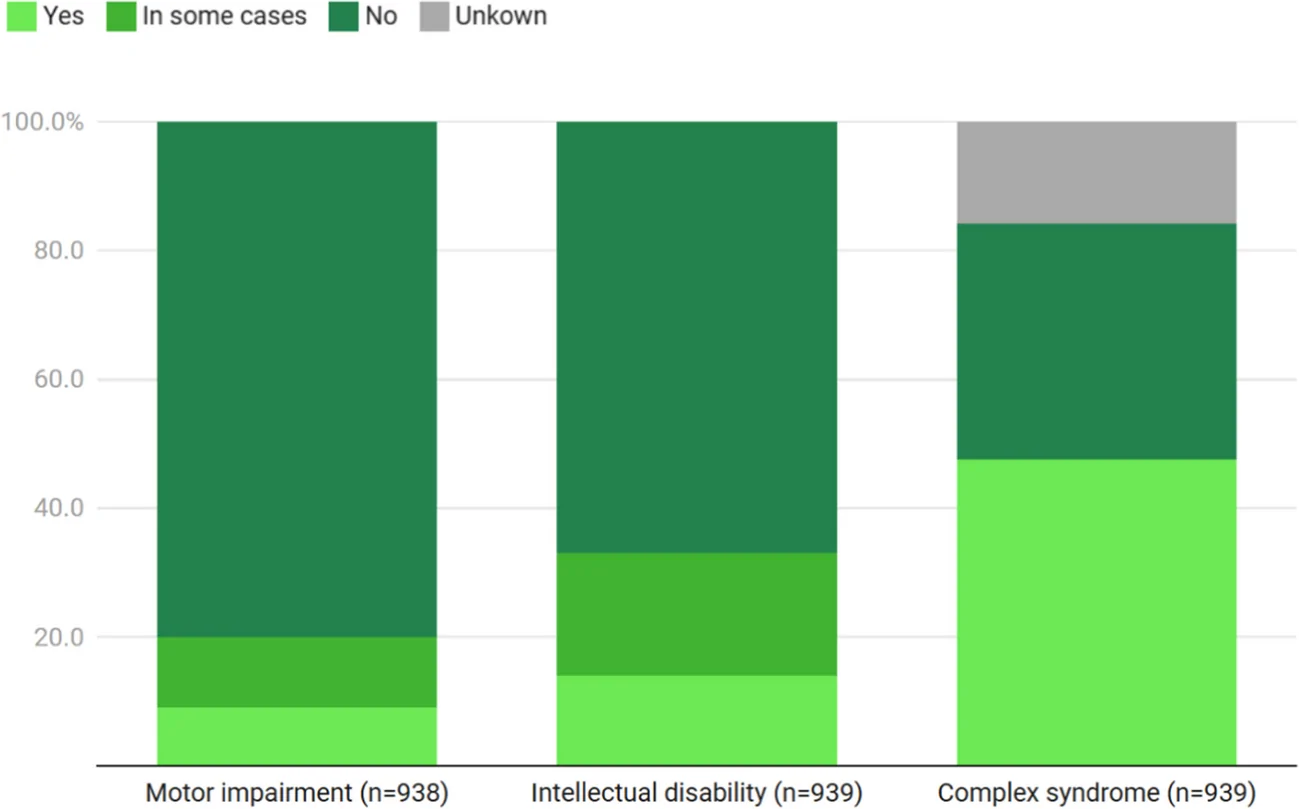
Percentage of respondents per clinician-based grouping. Note: Respondents may fall into multiple subgroups. Subgrouping was done by a clinician (MJS). Each grouping had three options *(always, in some cases* or *unknown*)

Based on the participants self-stated diagnosis the following subgrouping was made based on the symptoms of the diagnos (Fig. 2).

1. **Motor impairment** (*n* = 185): Only a small portion (19.7%, 185/938) have a diagnosis that commonly or always results in motor impairment.
2. **Intellectual disability** (*n* = 306): About a third of diseases commonly or always result in intellectual disability (32.6%, 306/940).
3. **Complex syndrome** (*n* = 448): Classification based on diagnosis impacting more than three organ systems (47.7%, 448/939). However, participants also self-reported whether or not their RD impacted more than one organ system (65.7%, 616/935).

Respondent-based subgrouping:

These additional subgrouping were based on respondents reporting:

4. **Especially short diagnostic journey** (Time to diagnosis < 6 months) (*n* = 344)
5. **Especially long diagnostic journey** (Time to diagnosis > 10 years) (*n* = 101)
6. **Large proportion of care with primary care** (More than half) (*n* = 204)
7. **Small proportion of care in primary care** (Less than half) (*n* = 326)
8. **Had contact with an expert in their RD** (*n* = 666)

This subgrouping was used for analysis of proportion of care within primary care, whether care needs are met, and trust for healthcare, HCPs, and primary care professionals, see below.

### Diagnosis journey

Most (99.7, 939/942) responded that they have a clinical diagnosis, though all 942 provided a specific diagnosis in the question asking what RD(s) they were diagnosed with.

### Age at diagnosis

The largest subgroup of respondents was diagnosed as adults (44.4%, 516/938), followed by during childhood (27.9%, 262/938) and at birth (16.7%, 157/938). The remaining were diagnosed during pregnancy, as a teen, “other”, or were unsure.

Respondents’ reported age of diagnoses was compared with the usual age of onset for their given disease as classified by clinician MJS (Table 3). Each specific disease was reviewed in regard to common symptoms, severity, and if any causative genes have been identified, using relevant scientific websites and databases (see methods). The timeframes generally aligned, with most respondents with a congenital disease having been diagnosed at birth, otherwise as a toddler, and the vast majority of those who would be expected to show symptoms in adulthood having been diagnosed in adulthood.

**Table 3:** Time to diagnosis by subgroup

Grouping/Labelling	Motor impair- ment *n* = 184 (%)	Intellec- tual disability *n* = 305 (%)	Complex syndrome *n* = 446 (%)	Over half of care in PC, *n* = 203 (%)	Under half of care in PC *n* = 325 (%)	All *N* = 939
Time to receive a diagnosis						
< 6 months	31.5	35.7	41.7	36	42.8	36.6
7–11 months	8.2	9.5	8.5	9.4	10.5	10.5
1–2 years	14.1	14.4	12.3	8.9	12.3	10.7
3–4 years	9.8	11.2	8.5	10.3	9.9	8.2
4–10 years	12	10.5	9.4	11.3	7.4	9.3
> 10 years	9.8	6.9	5.61	11.8	8	10.8
*Other* or* don’t know*	14.7	11.8	13.9	12.3	9.23	13.7

Table 3 presents the time to diagnosis (rows in %) by analysis grouping (columns). The analysis found that over one third of all groups were diagnosed in under six months (344/939, 36.6%) and another 10.5% within 7–11 months (99/939)

### Time to diagnosis

Of the responders, 36.6% (343/939) were diagnosed within 6 months from their first point of contact with healthcare. Additionally 100 individuals were diagnosed within 7–12 months, leading to that in total 47.2% (434 out of 939) were diagnosed within a year.

For 1 in 10 respondents, it took over 10 years to get their diagnosis (10.8%, 101/939). Table 3 presents the percentage diagnosed within a certain timeframe (columns) according to the usual age of onset (rows); the usual age of onset was determined by clinician MJS. The analysis found that respondents with a diagnosis typical of a younger age of onset tended to have a shorter diagnosis timeline.

### Genetic testing

**Fig. 3:**
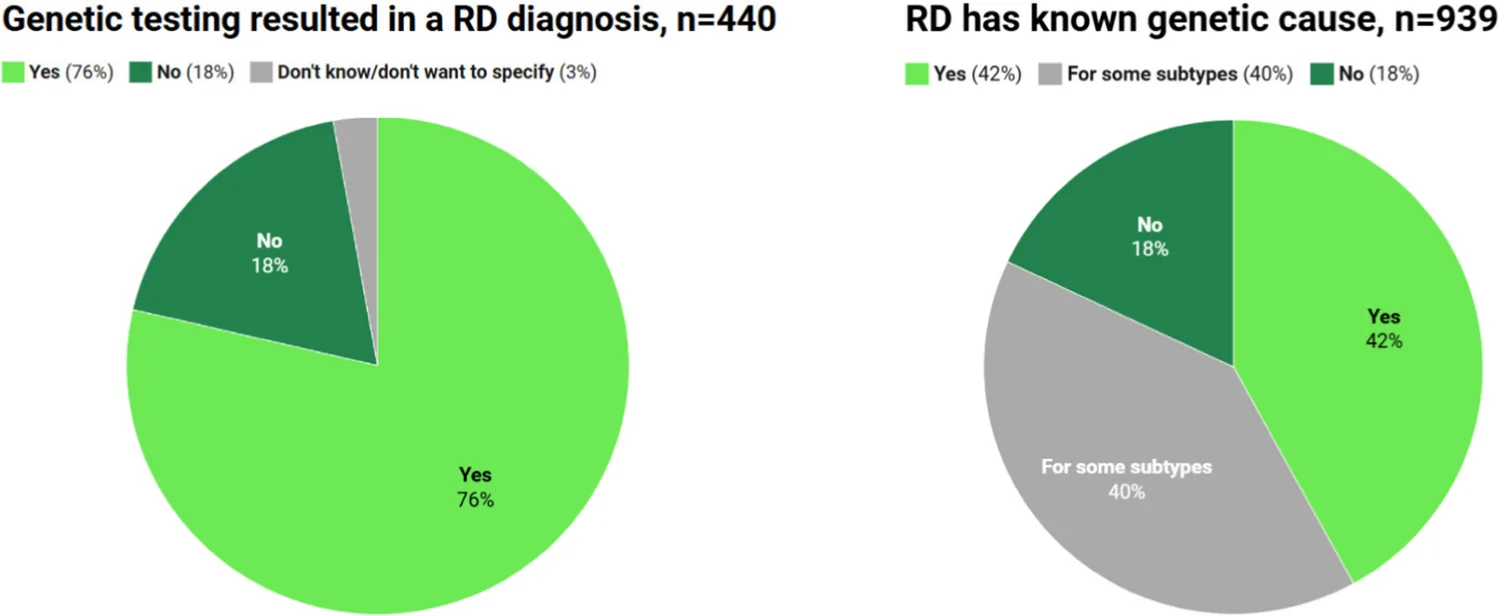
Percentage whose genetic testing resulted in a RD diagnosis (*n* = 440) and percentage whose RD diagnosis has a known genetic cause (*n* = 939)

According to the classification of whether the specific diagnoses have a known genetic cause, a large portion of RDs provided by respondents have a known genetic cause (82%, 770/939). However, only 446 of *all* respondents received genetic testing as part of the diagnosis process, which resulted in the diagnosis of one or more RDs for 76.1% (335/440), see Fig. 3. Again, considering MJS’ classification of whether or not the diagnosis has a known genetic cause, we found that 54.4% (419/770) of those (770) with a diagnosis with a known genetic cause underwent genetic testing. Of the small percentage for which there is no known genetic cause, 14.2% (24/169) reported having carried out genetic testing.

Of those who reported having been diagnosed with one or more RDs via genetic testing (*n* = 335), over a third (38.9%, 130/335) reported that they were diagnosed in childhood and roughly as many (38.3%, 128/335) were diagnosed in adulthood. The next largest subgroup (12.3%, 41/335) was diagnosed at birth.

Those who were not offered genetic testing (*n* = 443) were asked whether they would have liked it, of which 35% (155/443) reported that they did. Out of those 155, only (21.3%) 33 have a diagnosis with a known genetic cause. Additionally, half (49.8%, 77/155) of those individuals have a disease where genetic causes are known only for some subtypes.

### Disease impact

A large portion (39.2%, 361/921) spend an hour or less per week in healthcare or self-managing their RD(s). However, 19.5% (178/921) spend over five hours a week. Only about half (53.5%, 223/417) of P-PLWRD worked/studied full-time, of which 16.1% (64/397) reported that reduced hours were due to their role P-PLWRD to someone with a RD.

The majority (67.4%, 314/467) of those answering from the patient perspective reported that they work/study reduced hours due to their RD. The number of participants who reported working reduced work hours did not differ noteworthy between groups intellectual disability, motor impairment, complex syndrome and length of diagnosis journey.

### Healthcare use

Differences exist in how individuals’ healthcare is distributed and organized. Important variables are proportion of healthcare being provided by primary care, access to experts/specialists on the RD, and access to supporting services like care coordinators for increased continuity and coordination. An overview of reported distribution of these services is provided in Table 4.

**Table 4:** Utilization and experience of healthcare

	Count	%
Proportion of care in primary care, (*n* = 936)		
More than half	204	21.8
Mixed	164	17.6
Less than half	326	34.9
*Don’t know* or *Not relevant*	130	25.7
Number of care coordinators you have access to, (*n* = 937)		
Multiple	423	45.1
One	209	22.3
None, but I need one	131	14.0
None, and not needed	69	7.3
What is a care coordinator?	33	3.5
Not relevant	28	3.0
*Don’t know* or *Prefer not to specify*	69	7.4
Who is/are your care coordinator(s), (*n* = 634) *		
MD in primary care	207	32.7
MD in other specialty care	524	82.7
Nurse in primary care	75	11.8
Nurse in other specialty care	178	28.1
Psychologist	54	8.5
Social worker	69	10.9
Physiotherapist	184	29.0
Other	77	12.2
Usefulness of care coordinator(s), (*n* = 625)		
Yes, they are helpful in coordinating my care	228	36.5
No, they are not helpful in coordinating my care	158	25.3
*Don’t know*, *Not relevant* or *Prefer not to specify*	239	38.2

* Multiple-choice question, so percentages will not add up to 100%

**Fig. 4:**
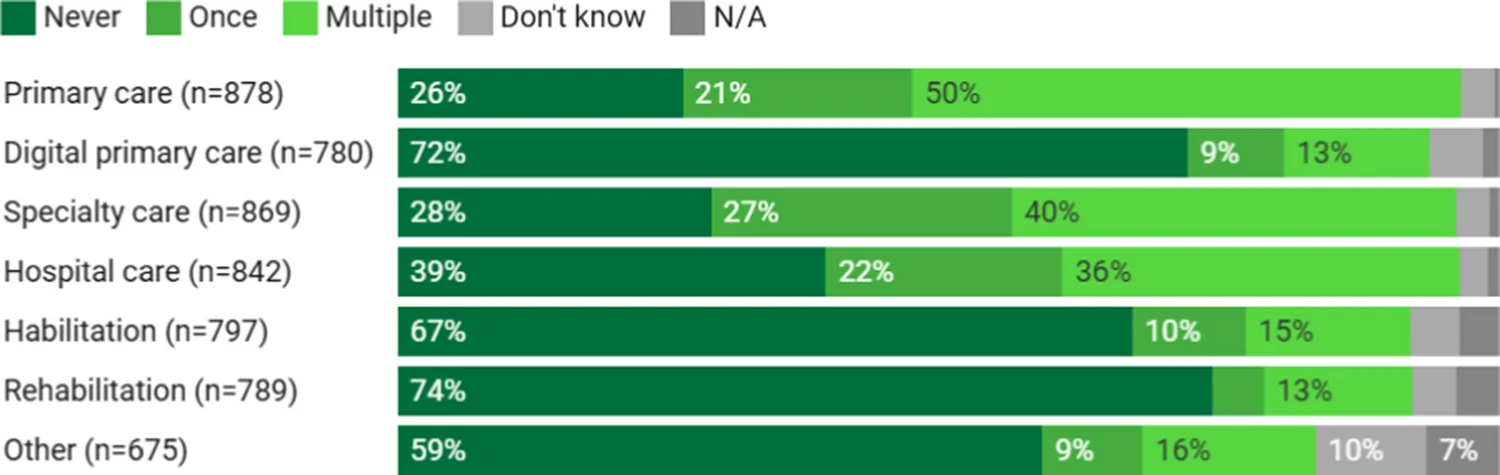
Percentage of frequency respondents visited each healthcare sector in the last 6 months

Respondents reported a high number of visits across multiple healthcare sectors (i.e. hospitals, PC), with 60–70% indicating at least one visit to each of the six sectors (in Fig. 4) over the past six months. Most visits were in-person primary care.

Overall, 70.6% (620/878) of participants reported one or more primary care visits, with rates differing by group: 65.6%, 275/419) among respondents living with a syndrome and 75.2% (130/173) among those with a motor disability. In contrast, very few digital visits were reported.

A minority (21.8%, 204/936) of respondents reported that their healthcare was mainly situated within primary care, see Table 4. This remains true within the individual levels of eight groupings (defined above e.g., motor impairment, intellectual disability, complex syndrome) as well (range 20%−24%). Chi-square tests of independence found a significant association between time to diagnosis and the proportion (under/over half, mixed) of care delivered in primary care, χ^2^(2, *N* = 337) = 15.21, *p* < 0.001, FDR-adjusted *p* = 0.026, Cramer’s *V* = 0.21. Those diagnosed after 10 years were more likely than expected to report mixed care (SR = 3.53) and less likely to report under half of care in PC (SR = –3.26). Respondents diagnosed within six months were more likely than expected to report under half of their care in primary care (SR = 3.26) and less likely to report mixed care (SR = –3.53). No pattern was found in relation to having a majority of care in PC.

**Fig. 5:**
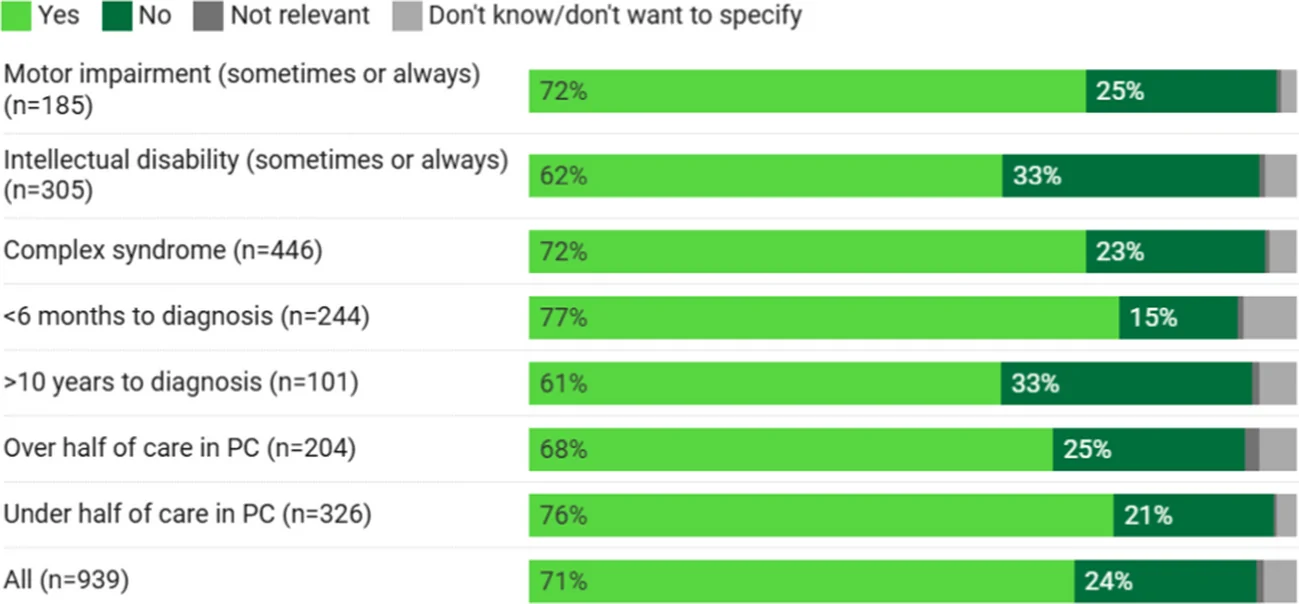
Consulted an expert on their RD

Seven out of ten reported that they have been in contact with an expert on their RD (at any given time). This number is the lowest among those with an intellectual disability (61.65%) and those with a long (> 10 year) diagnosis (61.39%). Most (139/204, 68.15%) of respondents with over half of care in PC had consulted an expert (Fig. 5), as had most (248/326, 76.07%) of those with less than half of care in PC. Although the association between expert contact and the proportion of care delivered in primary care reached nominal significance, χ^2^(2, *N* = 690) = 7.30, *p* = 0.03, it did not remain significant after false discovery rate correction (FDR-adjusted *p* = 0.52, Cramer’s *V* = 0.10). Nevertheless, respondents with an expert contact were more likely to report a minority (< 50%) of care within PC (SR = 2.65), whereas those without or unsure about expert contacts were less likely to have (SR = –2.65).

Less than half (47.5%) stated that they have a care coordinator with a HCP for continuity and/or help with coordinating care, of which the majority (82.7%) have a physician in a specialty other than primary care, and about a third have a primary care physician (32.7%), a nurse in another specialty (28.1%), and a physiotherapist (29%). A quarter (25.3%) of those with a care coordinator did not feel that their care coordination was helpful (Table 4).

### Perceptions of healthcare

Fewer than a third (29.9%, 280/936) responded that the level of knowledge within healthcare is sufficient to provide the correct treatment/follow-up for their RD, and just over half (51.4%, 481/936) responded that the knowledge within healthcare does not suffice. Only 11.7% (109/934) felt there is adequate support from healthcare and public services as they age, although the majority responded that they were unsure (59.3%, 554/934).

### Care needs met across groups

**Fig. 6:**
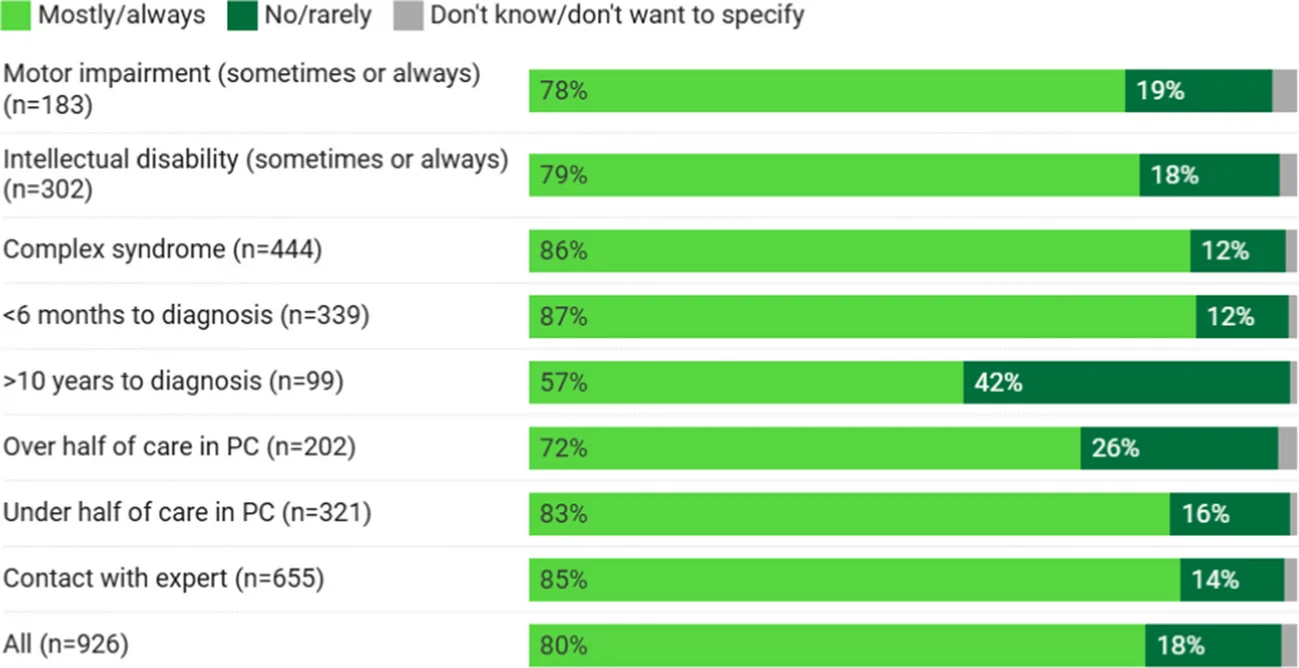
Whether care needs were met reported by group. *Note: Motor impairment and intellectual disability was categorized by a clinician and not by the respondent themselves. Syndrome is defined as a disease of three or more components.* > *10 years means from the first point of contact with health services regarding signs/symptoms associated with the diagnosis they eventually received*

Most participants (80.2%, 741/926) reported that their care needs were mostly or always met. The highest levels reported were by those who have met an expert in their RD (84.9%, 556/655). Around 80% (743/926) of all groups were mostly/always satisfied with their care. In contrast, 17.3% (163/926) indicated that their needs were not met (see Fig. 6).

The subgroup that reported the lowest satisfaction of care needs being met (56.6%, Fig. 6) was those who waited over 10 years for a diagnosis. These participants were diagnosed with a range of 41 different diagnoses, though four were listed more frequently, namely: mast cell diseases (*n* = 13), Poland’s syndrome, (*n* = 12), dystonia (*n* = 11), and Morbus Osler (HHT) (*n* = 10). Chi-square tests of independence found a strong association between diagnosis length and reported care needs, χ^2^(2, *N* = 438) = 45.94, *p* < 0.001, FDR-adjusted *p* = 0.013, Cramer’s *V* = 0.32. Respondents with a long diagnosis journey (> 10 years) were more likely than statistically expected to report unmet/rarely met needs (SR = 6.77) and less likely to report mostly/always met needs (SR = –6.59). Those diagnosed within 6 months showed the opposite pattern, and were more likely to report that their care needs were mostly/always met (SR = 6.59) and less likely to report unmet/rarely met (SR = –6.77).

There were also significant associations for having a complex syndrome, χ^2^(2, *N* = 923) = 17.90, *p* < 0.001, FDR-adjusted *p* = 0.009, Cramer’s *V* = 0.14, where respondents with a complex syndrome were more likely than expected to report that their care needs were met (SR = 4.23) and less likely to report their needs as unmet (SR = –3.97).

Respondents’ proportion of care within primary care was also significant, χ^2^(2, *N* = 523) = 10.65, *p* = 0.005, FDR-adjusted *p* = 0.01, Cramer’s *V* = 0.14. Respondents whose care was mostly within PC were overrepresented in reporting that their needs were not/rarely met (SR = 2.86) and underrepresented in reporting mostly/always (SR = − 3.20). The largest association was, however, whether respondents had seen an expert, χ^2^(2, *N* = 923) = 29.50, *p* < 0.001, FDR-adjusted *p* = 0.005, Cramer’s *V* = 0.18. Respondents with an expert contact were underrepresented in reporting that their care needs were not/rarely met (SR = − 5.07) and overrepresented in their needs being met (SR = 5.43). In contrast, those lacking an expert contact were overrepresented in reporting that their care needs were not/rarely met (SR = 5.43) and underrepresented in reporting their needs mostly/always met (SR = − 4.07).

No significant associations for motor impairment χ^2^(2, *N* = 922) = 1.81, *p* = 0.40, FDR-adjusted *p* = 0.45, Cramer’s *V* = 0.04. or intellectual disability χ^2^(2, *N* = 924) = 0.21, *p* = 0.90, FDR-adjusted *p* = 0.90, Cramer’s *V* = 0.01 were observed.

### Trust

**Fig. 7:**
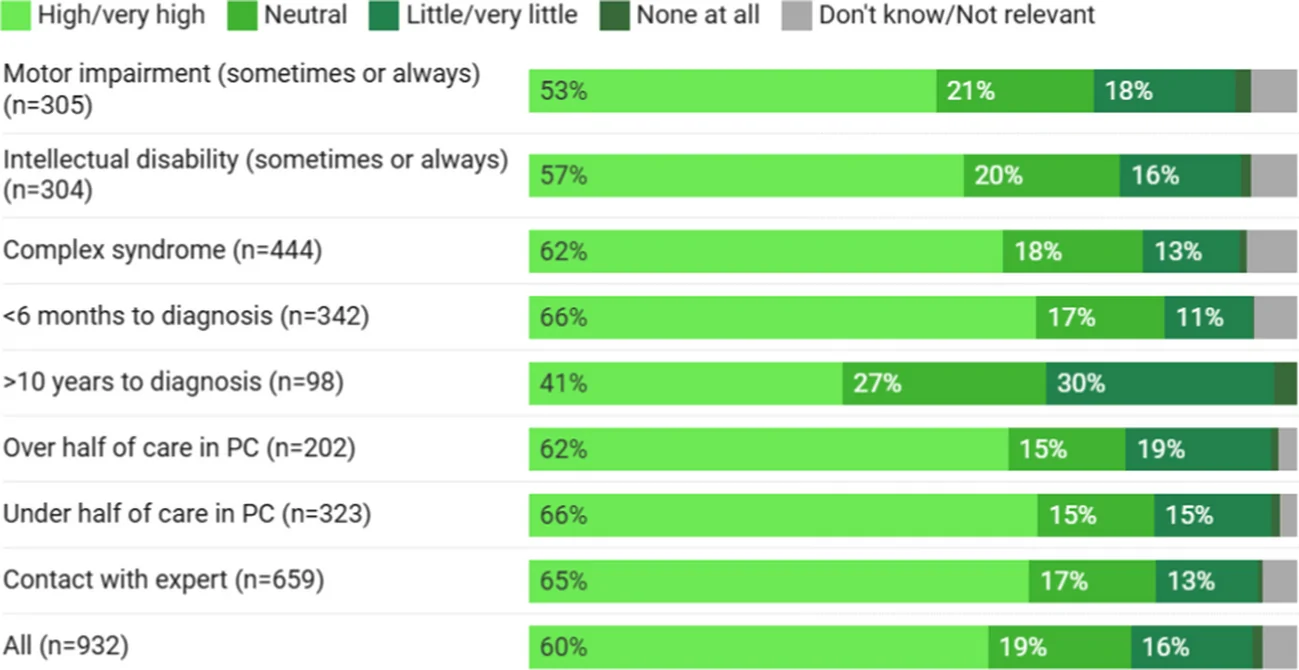
Trust in healthcare services reported by group

**Fig. 8:**
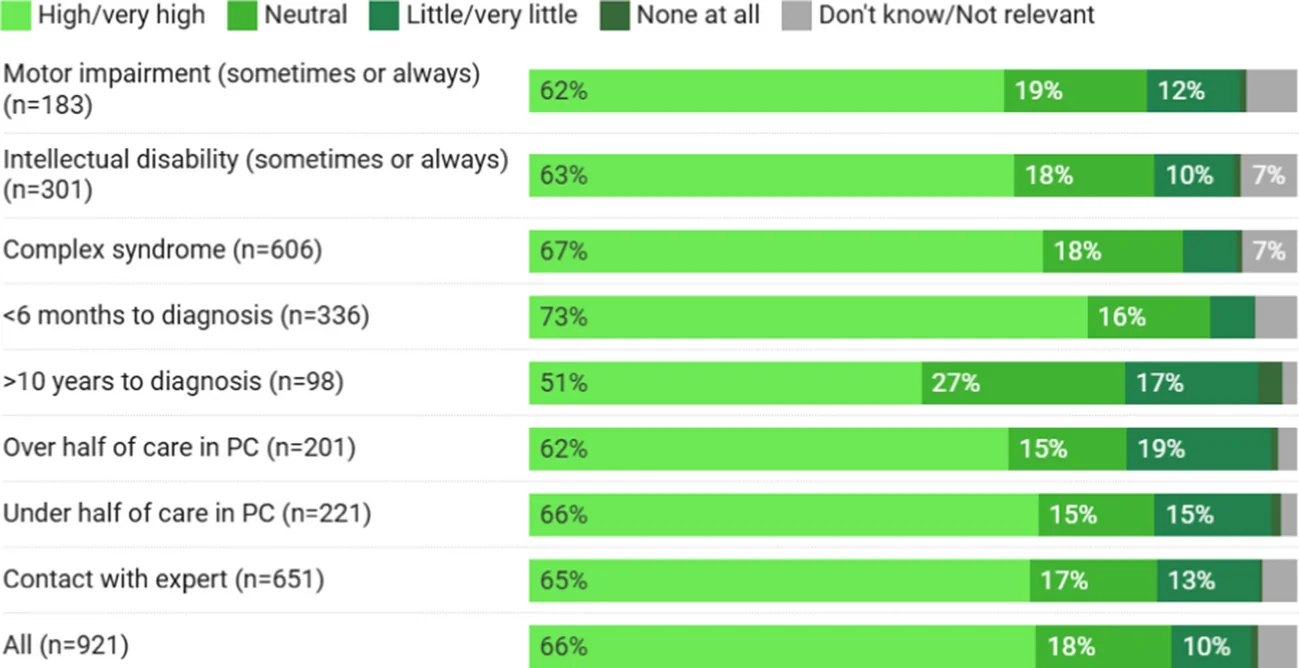
Trust in healthcare professionals reported by group

**Fig. 9:**
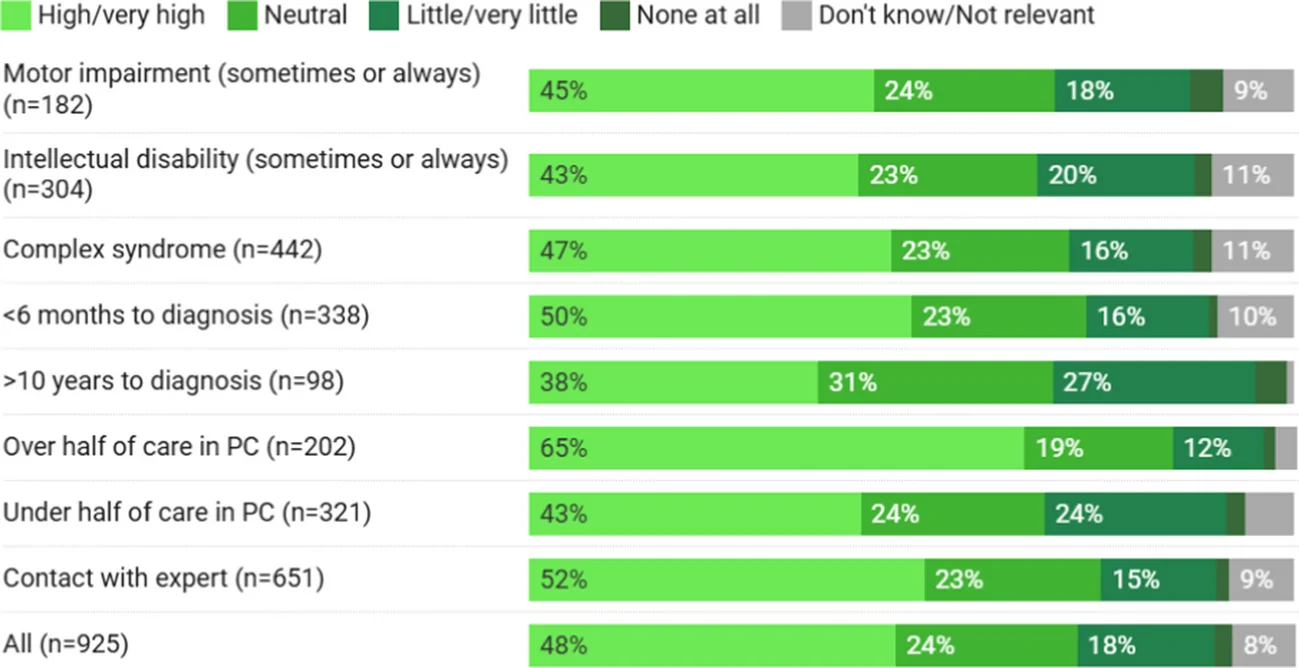
Trust in healthcare primary care professionals reported by group

Participants were asked to rate their level of trust in (1) healthcare services in general (2) healthcare professionals in general, and (3) primary care professionals (Figs. 7, 8, 9). Over half of respondents in all groups except those with a 10 + years diagnosis journey reported high/very high trust in services, and over half of all groups did for HCPs. In general, HCPs received higher trust than HC services.

#### Trust in healthcare (HC)

There was a significant association between having a complex syndrome and level of trust, χ^2^(4, *N* = 929) = 14.68, *p* = 0.005, FDR-adjusted *p* = 0.01, Cramer’s *V* = 0.13. Respondents with a diagnosis classified by our clinician as complex, were less likely than expected to report little/very little trust (SR = –2.57) and more likely to report don’t know/not relevant (SR = 2.82). Conversely, those where the classification of the diagnosis was less complex or unknown, respondents were more likely to report little/very little trust (SR = 2.47).

Time to diagnosis was also significant, χ^2^(4, *N* = 440) = 40.15, *p* < 0.001, FDR-adjusted *p* = 0.005, Cramer’s *V* = 0.30, indicating that a shorter diagnostic timeline was associated with higher trust. Respondents diagnosed within six months were overrepresented in high/very high trust category (SR = 4.51), whereas those diagnosed after 10 years were overrepresented in the little/very little trust category (SR = 4.39). Analysis was restricted to long vs short diagnosis groups; participants with medium-length diagnoses were excluded.

Finally, having had an expert contact showed the strongest association, χ^2^(16, *N* = 930) = 37.49, *p* < 0.001, FDR-adjusted *p* = 0.004, Cramer’s *V* = 0.20. Standardized residuals indicated that participants with an expert contact were more likely than expected to report high/very high trust in healthcare (SR = 5.15) and less likely to report little/very little trust (SR = –3.52) or neutral (SR = –2.52), whereas those who lacked an expert showed the opposite trend (SRs = –5.15, 3.33).

No significant associations were observed for motor impairment χ^2^(4, *N* = 928) = 5.62, *p* = 0.23, FDR-adjusted *p* = 0.331, Cramer’s *V* = 0.08, intellectual disability χ^2^(4, *N* = 930) = 3.69, *p* = 0.45, FDR-adjusted *p* = 0.532, Cramer’s *V* = 0.06, or proportion of care in primary care χ^2^(4, *N* = 525) = 1.40, *p* = 0.84, FDR-adjusted *p* = 0.874, Cramer’s *V* = 0.05.

#### Trust in healthcare professionals (HCPs)

In regard to HCPs, level of trust was significantly associated with time to diagnosis, χ^2^(4, *N* = 434) = 30.33, *p* < 0.001, FDR-adjusted *p* = 0.004, Cramer’s *V* = 0.26. Those diagnosed within 6 months were more likely than expected to report high/very high trust (SR = 4.02) and less likely than expected to report little/very little trust (SR = –3.69). In contrast, those with a long (> 10 years) diagnostic delay reported the opposite trend (SRs = –4.02, 3.69).

Respondents’ complex syndrome status (Yes vs. No/Unknown) was also significantly associated with reported trust, χ^2^(4, *N* = 918) = 16.22, *p* < 0.01, FDR-adjusted *p* = 0.019, Cramer’s *V* = 0.13. Standardized residuals indicated that participants without a complex syndrome were more likely than expected to report low/very low trust (SR = 3.06), whereas participants with a complex syndrome were more likely than expected to report low/very low trust (SR = –3.06).

The largest effect observed was, again, with having an expert contact (or not), χ^2^(4, *N* = 919) = 54.91, *p* < 0.001, FDR-adjusted *p* = 0.003, Cramer’s *V* = 0.24. Those *with* an expert contact were more likely than expected to report high/very high trust (SR = 6.29) and less likely than expected to report little/very little trust (SR = –4.69) or no trust at all (SR = –3.96). Those *without* an expert contact were more likely to report no (SR = 3.96) or little/very little trust (SR = 4.69) and less likely to report high trust (SR = –6.29).

Those *with* an expert contact were more likely than expected to report high/very high trust (SR = 6.29) and less likely than expected to report little/very little trust (SR = –4.69) or **no** trust at all (SR = –3.96). The opposite trend was found for those *without* an expert contact (SRs = –6.29, 3.96, 4.69).

No significant associations were found for motor impairment, χ^2^(4, *N* = 917) = 2.24, *p* = 0.69, FDR-adjusted *p* = 0.748, Cramer’s *V* = 0.05, intellectual disability, χ^2^(4, *N* = 919) = 4.46, *p* = 0.35, FDR-adjusted *p* = 0.455, Cramer’s *V* = 0.07, or whether participants received a majority or minority of care in primary care, χ^2^(4, *N* = 522) = 2.31, *p* = 0.69, FDR-adjusted *p* = 0.748, Cramer’s *V* = 0.07. No noteworthy deviations from expected cell counts were observed.

#### Trust in primary care professionals

The third form of trust evaluated was trust in primary care professionals. The proportion of care provided in primary care was significantly associated with trust in primary care professionals, χ^2^(20, *N* = 5230) = 24.59, *p* < 0.001, FDR-adjusted *p* = 0.003, Cramer’s *V* = 0.22. Those whose care was mostly in primary care were more likely than expected to report high/very high trust in primary care professionals (SR = 4.69) and less likely than expected to report having little/very little trust (SR = –3.34). In contrast, those with under half of care in primary care showed less likely than expected to trust (SR = –4.69) and reported more likely than expected to report low trust (SR = 3.34) in primary care professionals.

Time to diagnosis was associated with trust χ^2^(4, *N* = 436) = 20.13, *p* < 0.001, FDR-adjusted *p* = 0.003, Cramer’s *V* = 0.21, where those with a short diagnosis journey were more likely to report high/very trust (SR = 2.14) or don’t know/not relevant (SR = 2.90) and less likely to report little/very little trust for primary HCPs. (SR = –2.38). Respondents with a long diagnosis timeframe showed the inverse trend (SR = 2.38; –2.14; –2.90).

Contact with an expert was also significantly related to trust in primary care professionals, χ^2^(4, *N* = 923) = 23.24, *p* < 0.001, FDR-adjusted *p* = 0.002, Cramer’s *V* = 0.16. Those who had contact with an expert were more likely to report high/very high trust in primary care professionals (SR = 3.52) and less likely to report little/very little trust (SR = –3.66) or no trust at all (SR = –2.17). Conversely, those without an expert contact were more likely to report little/very little trust (3.66) and less likely to report high/very high trust (SR = –3.52).

While the raw *p*-values suggested nominal significance between having a complex syndrome and trust in HC services (χ^2^(4, *N* = 922) = 9.566, *p* = 0.05, Cramer’s *V* = 0.10), these associations were not significant after controlling for multiple comparisons (FDR-adjusted *p* = 0.081). There were no significant associations observed for intellectual disability, χ^2^(4, *N* = 923) = 7.82, *p* = 0.10, FDR-adjusted *p* = 0.153, Cramer’s *V* = 0.09, or motor disability, χ^2^(4, *N* = 921) = 4.63, *p* = 0.33, FDR-adjusted *p* = 0.331, Cramer’s *V* = 0.07.

## Discussion

The respondent subgroup included a balanced proportion of people living with a RD (PLWRD) answering for themselves, with and next of kin responding on behalf of an RD patient. This balance with the addition of those with a dual-role answering from both perspectives (PWLRD who is also next of P-PLWRD), offers a more comprehensive understanding of the family system affected by an RD. It has previously been shown that living within a family with RD has implications for both the diagnosed individual, the parents, siblings, and the family system as a whole (Atkins and Padgett 2024; Damen et al. 2022).

Our results show that even if almost half of individuals were diagnosed within a year from the start of symptoms, still one out of ten waited more than 10 years for a diagnosis (Table 3). The survey also found that the majority were diagnosed as an adult, and that a longer waiting time to diagnosis from the start of symptoms was experienced among adult-onset disorders (Table 2). Both prolonged diagnostic delays and having a higher proportion of healthcare within primary care were associated with less care needs met, as well as reduced trust in both healthcare providers and the healthcare system overall. However, these two groups partly overlap as having a greater proportion of the healthcare contacts within primary healthcare, seems to be correlated with longer waiting time for a diagnosis.

When comparing different disorder subgroups, we saw that those with a diagnosis associated with intellectual disability have less trust in primary healthcare services compared to the other subgroups; i.ethose having a complex syndrome and those having motor impairment) (Fig. 9). Individuals with disorders including motor impairment, on the other hand, reported less trust in health care services overall, compared to the other subgroups (Fig. 7). The individuals with an RD subgrouped as a complex syndrome indicated greater trust in the healthcare professionals than the other two groups (Fig. 8).

Among our respondents, having less than half of healthcare contacts in primary care or having a complex syndrome were both associated with a shorter time to diagnosis, A comparison between subgroups of those being diagnosed within 6 months from first contact, show that having less than half of your healthcare contacts in primary care and having a complex syndrome increases the chance for having a shorter time to diagnosis, whereas having primarily motor impairment or isolated IF, or more than half of your health care contacts in primary care or having motor impairment were both associated with a longer prolongs the time to diagnosis.

Our results are first to offer opportunities for looking deeper into which groups of PLWRD/P-PLWRD perceive most challenging and what factors are associated with diagnostic journeys and whichconsequenceslong waiting time for correct diagnosis have on individuals and their experience of healthcare.

Within our cohort, almost all reported that they/their next of kin had received a clinical diagnosis, and about one third had received a genetically verified diagnosis. Among the individuals who underwent a genetic test, four out of five received a genetic diagnosis. This is a higher level than the average diagnostic yield, which is approximately 20–40% (reviewed in Tesi et al. 2023) with today’s available genetic testing methods. This discrepancy could also be explained by the recruitment of respondents among the members of the patient organization Rare Diseases Sweden, as most people possibly become members after the diagnosis already is defined. Another explanation could be that the cohort of this survey may have gotten their clinical diagnosis in a more recent time, when genetic and genomic testing is more widely available. Another explanation could be that the individuals being associated with the Rare Disease Sweden association have a more severe and complex disease as they experience more challenges and therefore need more support.

The length of the diagnostic journey was investigated in depth as not having a diagnosis can delay and impair correct care, treatment, and support. With over 8000 RDs known today and diagnostic methods developing rapidly, this is a challenge for the HC system and patients. Long diagnostic journeys have been reported for people living with a rare disease (PLWRD) ranging between 5–7 years (Faye et al. 2024, Rare Diseases Sweden member survey 2024). In our cohort, the majority received a diagnosis within 6 months to 1 year from the first contact with healthcare. This is mainly among individuals with RDs with congenital onset who are diagnosed within six months in half of the cases, but the numbers drop to one third for onset as a toddler and varying onset, and to one fourth for onset in adulthood. In parallel, the number of individuals waiting for 4 years and more increases to about one fifth in the latter three groups. The results of Eurordis barometer also showed that those individuals whose symptoms start between the age of 10–19, had to wait more than ten years on average for a diagnosis. These results together with the results from our study show that long diagnostic journeys are associated with diseases with later onset in life. This could also partly be explained by that, congenital, newborn and childhood rare conditions are more often related to severe symptoms or being a syndrome, which could make it more “obvious” that it is a RD with a genetic cause, and therefore, a genetic test could be relevant for receiving a diagnosis, whereas adult-onset symptoms could be more subtle. Also, children are “automatically” followed at the child health centers (BVC) by routine protocols, for their development and thereby unusual development is more commonly detected. Adult individuals do not have that “automatic” follow-up system by healthcare and must take the responsibility of contacting healthcare themselves, mostly through primary care. Adults’ HC contacts are mostly in primary care, which does not have sufficient awareness, knowledge or established routines in their clinic to offer, and/or to refer for genetic/genomic testing; nor is it within their scope or economic resources within the Swedish HC organization (1177.se, Vårdgarantin 2024).

Another reason for adults receiving no or late diagnosis could be that, at the time when their symptoms started, the methods for genetic testing were limited, and no follow up for new genetic testing has been offered or performed since then. Resources in the clinical genetic setting are also limited, so there is no routine pathway for automatically offering new genetic/genomic tests, recontacting patients, or offering a reanalysis of data. Thus, the possibility of being offered to reanalyze data and recontact is more dependent on the patient contacting the healthcare system or on the specific doctors´ specific knowledge or update. Our results show that PLWRD have high HC needs, mirrored in the number of visits to HC services and amount of time invested in self-managing of their RD, both among care givers and PLWRD. Our results give valuable insight and incitement to investigate support needs that could free up resources among PLWRD or as P-PLWRD. Due to the complexity, and lack of standard procedures for treatment of PLWRD, handling care for this patient group requires high flexibility and is often time consuming, which usually is hard to achieve in primary HC. The time at the appointment is limited for each patient and the patient is usually given the possibility to treat only one symptom during each visit, making it challenging for a person with a complex condition. Our results confirm previous descriptions, with respondents with most of the care in primary HC reporting their health needs being met to the lowest degree. In contrast, respondents diagnosed within six months were less likely to have most of the care in primary HC. This population group also reported their health needs having been met to a higher degree.

Another possible reason that is mentioned in the Eurordis survey is that adult individuals might previously have been mis-diagnosed and because of receiving a diagnose (even if the wrong one) leads to not offering further tests until new symptoms or new suspicions of a specific diagnosis occur (Faye et al. 2024). This is however nothing that was explored in our survey.

Despite high levels of HC contact and health needs, only 70% have been in contact with an expert on their RD, the lowest being among those with intellectual disabilities (by expert we are referring to a HC professional with satisfactory knowledge about the RDs, their treatment and follow-up). At the same time, access to expertise is strongly associated with reports of health needs being met as well as with higher trust in the HC system and HC professionals. It is striking that more than two thirds of our cohort thinks that the knowledge about their or their child’s RD within healthcare is non-sufficient. In RDs, a specific expert/expert team is in many cases not available for most of the 8000 currently known RDs. Many times, there is no possibility to meet one expert covering all symptoms in a specific RD, but it is still possible they have met different healthcare providers with knowledge and expertise related to specific symptoms. International collaboration is therefore important. Current processes/work is being performed to better link international RD expertise and implement it into Swedish HC structures (European Reference Networks—Public Health—European Commission).

Of our study, most respondents, four out of five, state that their healthcare needs are met and over half state high trust in healthcare and healthcare providers. As above, individuals who met an expert have reported their healthcare needs met and felt trust in healthcare to the highest degree. However, there are subgroups of our cohort whose scores were lower than the overall majority. There are three main reasons for not having care needs met and feeling trust: Having a complex syndrome, waiting longer than 10 years for a diagnosis, and having most of their healthcare in primary care correlates to not having care needs met and to lower trust in healthcare and healthcare providers.

Thus, even the majority in our cohort, receive a diagnosis in a relatively short time, and only a small fraction has to wait more than 10 years for a diagnosis, it is here shown that decreasing time to diagnosis is of the highest importance for the individual, not only for achieving a diagnose to be able to have information on symptoms, prognosis and possible treatments, but in addition to help individuals to have their care needs met, and to increase their feeling of trust in healthcare.

In Sweden, support systems exist for people with complex health challenges and high HC needs. Tools like care coordinators (fast vårdkontakt) and multiprofessional patient rounds (MDT) are two examples which aim to improve treatment continuity and coordination of care. These solutions are legally anchored (The Swedish Patient Act (Patientlagen), but in reality, less than half of participants report having access. This disparity in care despite legal measures may partly be explained by regional organization in the Swedish healthcare system where each health care region still makes their individual prioritization based on regional resources and ethical concerns. Within our cohort, it was less common among the adult-onset respondents to have a care coordinator. This is in line with the healthcare organization in Sweden, where pediatric care has a more holistic approach to the child´s health problems and care needs whereas adult care is more prone to be “silos” meaning that for healthcare problems/symptoms from different organs are treated by separate specialties where communication between these is often lacking. Our results indicate that focused commitment to making these solutions easily available for the vulnerable group of PLWRD can have a large and positive impact for the individual and society. Optimizing HC processes and support for this population can help ease burden on the individual but also for the HC system.

Development of HC structures to improve HC for PLWRD therefore needs to focus on these patient populations and related HC services, maybe by primary healthcare centers focusing on RD patients allowing more support to the MDs and longer appointments for each individual when needed. In Sweden some regions already have these kind of centers, showing good results in meeting the healthcare needs for these patients (1177.se, Vårdgarantin 2024).

Even if Sweden is a small country with a relatively little population size, there is no national consensus on genomic healthcare, which due to the healthcare organization leads to inequality and injustice for the patients as it depends on where in the country they live. The 21 independent regions, with varying resources have the right to decide how to allocate them to genetic testing and specialty care. This leads to varying prioritization of and thereby injustice in (1) unequal access to genetic testing (2) access to treatment as to whether or not you have a genetically confirmed diagnosis (3) access to support from society on different levels (National Board of Health and Welfare 2025).

In many countries centers for RDs are built up, not only for advocating better care, but also meeting these persons for a holistic evaluation. To improve healthcare for RD patients, most countries in the European Union (EU), have a national strategy for RDs, (National plans or strategies for rare diseases: page updated—Public Health), but this is still lacking in Sweden. A strategy would give mandate to both authorities and healthcare providers to prioritize this patient population. Another example is EU supported ERN-networks that collect experts from all European countries and collaborate in multiprofessional teams (European Reference Networks—Public Health—European Commission). However, also in Sweden, several national initiatives aim to reduce inequalities and promote justice for patients, including the national strategy for rare diseases currently under development and Genomic Medicine Sweden (GMS; Genomic Medicine Sweden | Improved diagnostics, care and treatment), which seeks to reduce regional disparities in access to genomic healthcare within Sweden.

## Limitations

This survey presents perspectives of members of the *Rare Diseases Sweden*. As members, they are likely to have greater access to support services and health-related education compared to non-members. Consequently, people not included in this study may face even greater barriers in accessing and being satisfied with healthcare services. Additionally, it is possible that individuals who encounter difficulties or shortcomings within the healthcare system are more inclined to seek out such networks.

The majority of the respondents had a higher educational level (more than one third had a university/college degree), which might impact the generalizability of this study. However, this demographic pattern also mirrors the education level of the Swedish population (Statistiska Centralbyrån). The high educational level of the respondents could also be due to the fact that the survey only reached out to members of the patient organization “Rare diseases Sweden”, and that individuals with higher education are associated with higher health literacy, higher capability of completing extensive surveys and greater health-engagement behaviors, and that is why individuals with higher education are more prone to fill in surveys and to engage with patient organizations in general (Rosário et al 2024). Additionally, as the survey was quite extensive, it could lead to higher educated individuals responding to a higher degree, all resulting in a bias of respondents. Consequently, the results presented here may overestimate the quality of care for PLWRD in Sweden as educational level, health literacy and access to, and use of, high quality healthcare are correlated (Rosário et al. 2024). Therefore our results might not be generalizable to a broader cohort of the rare disease community. Data was collected at the end of 2021, coming out of the COVID-19 pandemic, meaning that any influence the pandemic had would likely be reflected in the responses. Another aspect which may have changed for some RDs, though not all, is clinical knowledge, guidelines, and standard diagnosis/treatment (e.g. new genetic testing).

The subgroup labelling of respondents according to the diagnosis was done by a clinician using available data and referencing relevant clinical websites. Since there was no data available from the PLWRD’s medical journals and many RDs can have a very variable clinical picture, some respondents might have been mislabeled regarding symptoms, as the subgrouping is made on a general picture of the manifestations of the disorder. Also, we have excluded all respondents that have not specified their diagnosis. Survey questions were skippable, and answer options “not relevant,” “don’t know” and “prefer not to specify” meant that for some questions, too few responses were collected to draw any conclusions.

## Conclusion

Unexpectedly, half of the PLWRD in our study received a diagnosis within a year, and a majority of them reported high levels of care needs met and healthcare thrust. However, the subgroup that had to wait over 10 years to receive a diagnosis showed low trust in healthcare, and low levels of care needs met. Also, not having met an expert was associated with low levels of care needs met and trust in healthcare. As two out of three experienced insufficient knowledge among healthcare providers about their RD, it is of great importance that awareness, knowledge and education related to RDs, as well as to genetics/genomics, will increase in all healthcare sectors, including primary care as many people living with RD have a great part of their HC in primary care. It is also of great importance to invest in diagnostics and development of, as well as availability, to genomic testing. Results from our study need to be considered when decisions are being made about allocation of national resources, including a national strategy, to support PLWRD into the Swedish HC system.

## Supplementary Information

Below is the link to the electronic supplementary material.

Supplementary file1 (PDF 292 KB)

Supplementary file2 (DOCX 34 KB)

Supplementary file3 (DOCX 1642 KB)

## Data Availability

The raw data from the survey responses can be obtained via an Excel file if requested.
