# Supplementary material for: Diagnostic delay, care needs, and trust in healthcare among persons with rare disease and their next of kin living in Sweden
Source: J Community Genet. 2026 Aug 20;17(5):102. doi: 10.1007/s12687-026-00931-6 (PMC13493750; doi:10.1007/s12687-026-00931-6)
Supplement: Supplementary file 1 [file 12687_2026_931_MOESM1_ESM.pdf]

SÄLLSYNTA DIAGNOSER
RARE DISEASES SWEDEN

Riksförbundet Sällsynta diagnoser - medlemsundersökning hösten 2021

Riksförbundet Sällsynta diagnoser arbetar för nationell samordning av vården för sällsynta hälsotillstånd. Tack för att du tar dig tid att svara på denna undersökning då det är till stor nytta i vårt påverkansarbete.

I år har även följande aktörer bidragit med frågor till vår undersökning: Ågrenska, Centrum för Sällsynta Diagnoser i Samverkan, Myndigheten för vård- och omsorgsanalys, Statens beredning för medicinsk och social utvärdering (SBU), Tandvårds- och Läkemedelsförmånsverket (TLV), Kompetenscenter för sällsynta odontologiska tillstånd och de forskande läkemedelsföretagen (Lif).

Svaren samlas in helt anonymt och ingen kommer kunna se vem som svarade vad. Resultaten analyseras på gruppnivå av oss på Riksförbundet tillsammans med undersökningsföretaget Stoswe och kommer att förmedlas till våra samarbetspartners.

Formuläret är omfattande och det kommer uppskattningsvis att ta mellan 30 och 60 minuter beroende på hur många fritextsvar du väljer att fylla i. I fritextsvar kan du skriva upp till 360 tecken. Du behöver inte svara på alla frågor på en gång utan det går bra att avbryta svarandet, spara dina svar och fortsätt vid senare tillfälle.

Etikprövningsmyndigheten
2023-06900-01-435533
2023-11-06

Är du som fyller i enkäten yngre än 16 år behöver du göra det tillsammans med en vuxen person.

Har du frågor eller funderingar hör gärna av dig till info@sallsyntadiagnoser.se.

Så fyller du i enkäten:

Svara på frågorna genom att markera något eller några av alternativen. Flera frågor är öppna så du kan kommentera med egen text.

När du är klar på en sida klickar du på "**Nästa**" längst ner till höger för att gå vidare till nästa sida.

Det går att backa i undersökningen genom att klicka på "Föregående" längst ner till vänster. Du kan även fortsätta att svara på undersökningen vid senare tillfälle genom att klicka på "Spara och fortsätt senare" nere till vänster och därefter följa instruktionerna.

När du fyllt i hela enkäten avslutar du genom att trycka på "**Skicka**" längst ner till höger på sidan.

1. Lever du själv med ett sällsynt hälsotillstånd, eller är du närstående med en eller flera personer som lever med sällsynta hälsotillstånd?

Om du är både och, behöver du bestämma dig nedan om du vill fylla i

enkäten för dig själv eller en av dina närstående. Det är viktigt att du svarar på alla frågor i hela enkäten från samma utgångspunkt, som en och samma person.

- ◯ Jag är närstående till en eller flera personer som lever med ett sällsynt hälsotillstånd
- ◯ Jag lever själv med ett sällsynt hälsotillstånd
- ◯ Jag lever själv med ett sällsynt hälsotillstånd **och** som närstående till en eller flera personer och jag fyller i enkäten **för mig själv**
- ◯ Jag lever själv med ett sällsynt hälsotillstånd **och** som närstående till en eller flera personer och jag fyller i enkäten för **en av mina närstående**
- ◯ Vill inte ange

Etikprövningsmyndigheten
2023-06900-01-435533
2023-11-06

2. Min relation till personen som lever med ett sällsynt hälsotillstånd är att jag är:

- ◯ Syskon
- ◯ Förälder
- ◯ Barn
- ◯ Partner
- ◯ Annan släkt
- ◯ Annan närstående
- ◯ Annat, nämligen ________
- ◯ Vill inte ange

3. Du som är närstående är

- ◯ Kvinna
- ◯ Man
- ◯ Annat
- ◯ Vill inte ange

4. Du som är närstående, hur gammal är du?

- ○ Yngre än 18 år
- ○ 19-24 år
- ○ 25-34 år
- ○ 35-44 år
- ○ 45-54 år
- ○ 55-64 år
- ○ 65-74 år
- ○ 75 år eller äldre
- ○ Föredrar att inte ange ålder

Etikprövningsmyndigheten
2023-06900-01-435533
2023-11-06

5. Vilken utbildningsnivå har du som är närstående?

- ○ Grundskola
- ○ Grundsärskola
- ○ Gymnasieskola
- ○ Gymnasiesärskola
- ○ Universitet/högskola
- ○ Annan utbildning
- ○ Vill inte ange

6. Du som är närstående, i vilket land är du född?

- Sverige
- Övriga Norden
- Övriga Europa inklusive Ryssland och Turkiet
- Övriga världen
- Vill inte ange

7. I vilken omfattning arbetar eller studerar du som är närstående?

- 100%
- 75%
- 50%
- 25%
- Ingen anställning och studerar inte
- Vilil inte ange
- Annat

Etikprövningsmyndigheten
2023-06900-01-435533
2023-11-06

8. Arbetar eller studerar du som är närstående i mindre utsträckning än 100% på grund av det sällsynta hälsotillståndet?

- Ja. Förtydliga vid behov
- Nej
- Kanske
- Vill inte ange

Alla frågor som följer nedan ber vi dig besvara ur perspektivet av den som lever med ett sällsynt hälsotillstånd. Är du närstående och har fler än en närstående med sällsynt hälsotillstånd bestäm dig för en person.

Frågorna nedan handlar om bakgrunden för personen som lever med det sällsynta hälsotillståndet.

9. Vilket kön har du/din närstående som lever med ett sällsynt hälsotillstånd?

- ◯ Kvinna
- ◯ Man
- ◯ Annat
- ◯ Vill inte ange

10. Hur gammal är du/din närstående med ett sällsynt hälsotillstånd?

- ◯ 6 år eller yngre
- ◯ 7-11 år
- ◯ 12-18 år
- ◯ 19-24 år
- ◯ 25-34 år
- ◯ 35-44 år
- ◯ 45-54 år
- ◯ 55-64 år
- ◯ 75 år eller äldre
- ◯ Föredrar att inte ange ålder
- ◯ 65-74 år

11. Vilken är din/din närståendes högsta utbildningsnivå?

- ◯ Går i förskola/grundskola
- ◯ Grundskola
- ◯ Grundsärskola
- ◯ Gymnasieskola

Etikprövningsmyndigheten
2023-06900-01-435533
2023-11-06

- ◯ Gymnasiesärskola
- ◯ Universitet/högskola
- ◯ Vill inte ange
- ◯ Annan utbildning ______

12. I vilket land är du/din närstående som lever med ett sällsynt hälsotillstånd född?

- ◯ Sverige
- ◯ Övriga Norden
- ◯ Övriga Europa inklusvie Ryssland och Turkiet
- ◯ Övriga världen
- ◯ Vill inte ange

Etikprövningsmyndigheten
2023-06900-01-435533
2023-11-06

13. Vilken region bor du/din närstående i? Vi ställer denna fråga för att kunna jämföra olika regioner men du förblir anonym.

- ◯ Landstinget Sörmland
- ◯ Region Blekinge
- ◯ Region Dalarna
- ◯ Region Gotland
- ◯ Region Gävleborg
- ◯ Region Halland
- ◯ Region Jämtland Härjedalen
- ◯ Region Jönköping
- ◯ Region Kalmar län
- ◯ Region Kronoberg
- ◯ Region Norrbotten
- ◯ Region Skåne

- Region Stockholm
- Region Uppsala
- Region Värmland
- Region Västerbotten
- Region Västernorrland
- Region Västmanland
- Region Örebro län
- Region Östergötland
- Västra Götalandsregionen
- Utomlands
- Vill inte ange

Etikprövningsmyndigheten
2023-06900-01-435533
2023-11-06

Frågorna nedan handlar om det sällsynta hälsotillståndet och diagnosen.

14. Har du/din närstående med det sällsynta hälsotillståndet en eller flera fastställda, sällsynta diagnoser?

- Ja, en eller flera sällsynta diagnoser som står med i min patientjournal
- Ja, en eller flera sällsynta diagnoser men **inte alla** står med i min patientjournal
- Ja, en eller flera sällsynta diagnoser men **ingen** står med i min patientjournal
- Nej, jag lever med ett sällsynt hälsotillstånd men har ingen fastställd diagnos
- Vet inte
- Vill inte ange

15. Vilken eller vilka diagnoser har du/din närstående? Ange nedan. Du kan välja flera svarsalternativ.

- [ ] Angelmans syndrom
- [ ] Alströms syndrom
- [ ] Atypiskt hemolytiskt uremiskt syndrom (aHUS)

- [ ] Aicardis syndrom
- [ ] Aorta Dissektion
- [ ] Akromegali
- [ ] Anonymous
- [ ] Artrogryposis Multiplex Congenita (AMC)
- [ ] Alströms syndrom
- [ ] Addisons sjukdom (Morbus Addison)
- [ ] Aniridi
- [ ] Aperts syndrom
- [ ] Alports syndrom
- [ ] BIH Pseudotumor cerebri: PTC
- [ ] Bethlems sjukdom LGMD-typ 5
- [ ] Beckwith-Wiedemanns syndrom
- [ ] Curry-Jones Syndrom
- [ ] Cavernoma, Kavernom, Kavernöst Angiom/Malformation
- [ ] Costellos syndrom
- [ ] CAP Myopati
- [ ] Coffin-Lowrys syndrom
- [ ] Cornelia de Langes syndrom
- [ ] Capillary leak syndrom
- [ ] Cushings syndrom
- [ ] CATCH 22/22q11-deletionssyndrom
- [ ] Congenital adrenal hyperplasia (CAH)
- [ ] Dystoni
- [ ] DIDMOAD
- [ ] Dandy-Walkers syndrom
- [ ] Dravets syndrom
- [ ] Dysmeli

Etikprövningsmyndigheten
2023-06900-01-435533
2023-11-06

- [ ] Erytromelalgi
- [ ] Ehlers-Danlos syndrom (EDS)
- [ ] Ektodermal Dysplasi (ED)
- [ ] Epidermolysis Bullosa (EB)
- [ ] Feingold syndrom
- [ ] Familjär amyloidos med polyneuropati – skelleftesjukan
- [ ] Fabrys sjukdom
- [ ] Feokromocytom
- [ ] Fibrodysplasia Ossificans Progressiva (FOP)
- [ ] Fragile-X-syndromet (FRAXA)
- [ ] Friedreichs ataxi
- [ ] Freeman-Sheldon syndrom
- [ ] Galaktosemi
- [ ] Huntingtons sjukdom
- [ ] Hereditära multipla osteokondrom
- [ ] Heterotaxi syndrom (Isomerism)
- [ ] Hyperkalemisk periodisk paralys
- [ ] Hyperexplexia
- [ ] Herpes simplexencefalit
- [ ] Hypoparathyroidism
- [ ] Hypofysinsufficiens
- [ ] Hereditär spastisk parapares (HSP)
- [ ] Hemparenteral nutrition (HPN)
- [ ] IIH Benign intracranial hypertension
- [ ] Idiopatisk Intrakraniell hypertension (IIH)
- [ ] Infantil Tay Sachs
- [ ] Indolent systemisk mastocytos
- [ ] Iktyos

Etikprövningsmyndigheten
2023-06900-01-435533
2023-11-06

- [ ] Kongenitala muskeldystrofier
- [ ] Klippel Feils syndrom
- [ ] Kausalgi
- [ ] Kraniofaryngiom
- [ ] Kallmans syndrom
- [ ] Kennedys sjukdom
- [ ] Kraniofaciala missbildningar
- [ ] Klinefelters syndrom
- [ ] Lymfödem
- [ ] Loeys-Dietz syndrom (LDS)
- [ ] Lymphoedema congenital
- [ ] Linjär Sklerodermi
- [ ] Lymfangioleiomyomatos (LAM)
- [ ] Laurence-Moon-Bardet-Biedls syndrom (LMBB)
- [ ] Lebers hereditära optikusneuropati (LHON)
- [ ] Leukodystrofi (MLD)
- [ ] Morbus Osler (HHT)
- [ ] Makrofagisk Myofacit (MMF)
- [ ] Marshall Smith Syndrome (MSS)
- [ ] Multipel epifysär (epifyseal) dysplasi (MED)
- [ ] Mitokondriella sjukdomar
- [ ] Mastcellssjukdomar
- [ ] Milroys sjukdom (Lymfödem)
- [ ] Marfans syndrom
- [ ] Morbus Gaucher
- [ ] Mucopolysackaridos sjukdom (MPS)
- [ ] Möbius syndrom
- [ ] Mc Ardles sjukdom

Etikprövningsmyndigheten
2023-06900-01-435533
2023-11-06

- [ ] Multipel System Atrofi (MSA)
- [ ] Neuromyelitis Optica (NMO)
- [ ] Nefrogen diabetes insipidus
- [ ] Noonans syndrom
- [ ] Neurofibromatos (NF), typ 1 och 2
- [ ] Osteogenesis imperfecta (OI)
- [ ] Odiagnostiserade hjärnsjukdomar
- [ ] PBC – Primär biliär cholangit
- [ ] Pitt-Hopkins syndrom (PTHS)
- [ ] Parry-Romberg syndrom
- [ ] Primärt hereditärt lymfödem typ 1
- [ ] Pontine tegmental cap dysplasia (PTCD)
- [ ] PKU (Föllings sjukdom)
- [ ] Prader-Willis syndrom (PWS)
- [ ] Paroxysmal nokturn hemoglobinuri (PNH)
- [ ] Perisylvärt syndrom (BPP, CBPS)
- [ ] Polands syndrom
- [ ] Porfyrisjukdom
- [ ] Primärt antifosfolipidsyndrom (APS)
- [ ] Retts syndrom
- [ ] Septo-optisk dysplasi
- [ ] Spinal muskelatrofi typ 2 (SMA II)
- [ ] Spinal Muskelatrofi typ 3 (SMA III)
- [ ] Silver-Russels syndrom
- [ ] Spinocerebellär ataxia (SCA)
- [ ] Smith-Magenis Syndrom
- [ ] Syringomyeli
- [ ] Spielmeyer-Vogts sjukdom

Etikprövningsmyndigheten
2023-06900-01-435533
2023-11-06

- ☐ Sotos syndrom
- ☐ Sturge Weber syndrom
- ☐ Transversell myelit/idiopatisk myelit (TM)
- ☐ Trombotisk trombocytopen Purpura (TTP)
- ☐ TAR-syndromet
- ☐ Tuberös Skleros Complex (TSC)
- ☐ Turners syndrom (TS)
- ☐ VACTERL-associationen
- ☐ Wilsons sjukdom
- ☐ Williams syndrom
- ☐ Wolfram syndrom (DIDMOAD)
- ☐ XLH
- ☐ Vill inte ange
- ☐ Annan, nämligen ____________

Etikprövningsmyndigheten
2023-06900-01-435533
2023-11-06

16. När fastställdes den sällsynta diagnosen?

- ○ Under graviditeten
- ○ Vid födseln
- ○ Under barnaåren
- ○ Under tonårstiden
- ○ Som vuxen
- ○ Vet inte
- ○ Vill inte ange
- ○ Annat nämligen ____________

17. Hur lång tid tog det att få den sällsynta diagnosen från att första kontakt togs med vården?

○ Mindre än 6 månader

○ 7-11 månader

○ 1-2 år

○ 3-4 år

○ 4-10 år

○ Mer än 10 år

○ Vet inte

○ Vill inte ange

○ Annat ____________

18. Är det sällsynta hälsotillståndet du/din närstående lever med ett syndrom, dvs. att det kan ha effekt på mer än ett organsystem?

Etikprövningsmyndigheten
2023-06900-01-435533
2023-11-06

○ Ja

○ Nej

○ Vet inte

○ Vill inte ange

○ Ej relevant

19. Är du du/din närstående medlem i en diagnosförening eller ingår du i Riksförbundet Sällsynta diagnosers fria grupp (för medlemmar som saknar diagnosförening)?

○ Medlem i diagnosförening

○ Medlem i fria gruppen

○ Ej medlem i en diagnosförening

○ Vet inte

○ Annat ____________

Frågorna nedan handlar om din/din närståendes hälsa.

20. Hur skulle du uppskatta din/din närståendes hälsa?

1

God hälsa [slider] Dålig hälsa

1 10

21. Om du inte uppskattar din/din närståendes hälsa som god, ange gärna varför nedan.

500 tecken kvar

Etikprövningsmyndigheten
2023-06900-01-435533
2023-11-06

22. Vad är viktigast för dig/din närstående för att kunna ha en god hälsa?

500 tecken kvar

23. När du/din närstående har haft behov av läkarvård eller annan sjukvård; anser du/din närstående att du har fått det?

- ◯ Ja, alltid
- ◯ Ja, oftast
- ◯ Nej, enstaka gånger har jag inte fått läkarvård eller annan sjukvård vid behov
- ◯ Nej, det är regelbundet att jag inte får läkarvård eller annan sjukvård vid behov
- ◯ Vet inte
- ◯ Vill inte svara på frågan

24. Vad tror du är orsaken till att du/din närstående inte har fått läkarvård eller annan sjukvård när du/hen haft behov av det?

500 tecken kvar

Frågorna nedan handlar om genetisk testning.

25. Har du/din närstående blivit tillfrågad att göra en genetisk testning för det sällsynta hälsotillståndet?

- Ja
- Nej
- Vet inte
- Vill inte ange

Etikprövningsmyndigheten
2023-06900-01-435533
2023-11-06

26. Har du/din närstående genomgått en genetisk testning för det sällsynta hälsotillståndet?

- Ja
- Nej
- Vet inte
- Vill inte ange

27. Hade du/din närstående velat göra en genetisk testning för det sällsynta hälsotillståndet?

- Ja
- Nej
- Vet inte

◯ Vill inte ange

28. Resulterade det genetiska testet i en eller flera sällsynta diagnoser?

◯ Ja

◯ Nej

◯ Vet inte

◯ Vill inte ange

29. Upplever du/din närstående att bemötandet och handläggning inom vården, relaterat till det sällsynta tillståndet, har förändrats sedan diagnosen ställdes?

◯ Ja. (Ge gärna exempel) [ ]

◯ Nej

◯ Vet inte

◯ Vill inte ange

Etikprövningsmyndigheten
2023-06900-01-435533
2023-11-06

30. Upplever du att du/din närstående fick tillräcklig information inför den genetiska testningen, så kallad genetisk vägledning?

◯ Ja

◯ Nej

◯ Vet inte

◯ Vill inte ange

31. Av vem fick du/din närstående information/genetisk vägledning inför den genetiska testningen? Du kan kryssa i flera svarsalternativ.

☐ Läkare inom primärvården

- [ ] Sjuksköterska inom primärvården
- [ ] Läkare inom specialistvård
- [ ] Sjuksköterska inom specialistvård
- [ ] Läkare inom klinisk genetik
- [ ] Sjuksköterska/genetisk vägledare inom klinisk genetik
- [ ] Ingen har informerat mig inför den genetiska testningen
- [ ] Vill inte ange
- [ ] Vet inte
- [ ] Annan

32. Upplever du att du/din närstående fick bra information/genetisk vägledning kring resultatet efter den genetiska testningen?

- ○ Ja
- ○ Nej
- ○ Vet inte
- ○ Vill inte ange

Etikprövningsmyndigheten
2023-06900-01-435533
2023-11-06

33. Av vem fick du information/genetisk vägledning kring resultatet efter den genetiska testningen? Du kan välja flera svarsalternativ.

- [ ] Läkare inom primärvården
- [ ] Sjuksköterska inom primärvården
- [ ] Läkare inom specialistvård
- [ ] Sjuksköterska inom specialistvård
- [ ] Läkare inom klinisk genetik
- [ ] Sjuksköterska/genetisk vägledare inom klinisk genetik
- [ ] Jag har enbart fått resultat, ej genetisk vägledning efter den genetiska testningen
- [ ] Vet inte

☐ Vill inte ange

☐ Annan

Nedan följer frågor som rör det vardagliga livet. Du som är närstående kom ihåg att besvara frågorna ur perspektivet av den som lever med ett sällsynt hälsotillstånd.

34. Hur påverkar ditt/din närståendes sällsynta hälsotillstånd vardagen? Dina fritextsvar kommer oavkortat förmedlas till myndigheten SBU (statens beredning för medicinsk och social utvärdering).

500 tecken kvar

Etikprövningsmyndigheten
2023-06900-01-435533
2023-11-06

35. Uppskatta hur mycket tid du/din närstående lägger ner i genomsnitt per vecka på vård och administration av det sällsynta hälsotillståndet (tex egenvård, kontakter med sjukvård, myndigheter, samhällsservice, med mera)

○ 0-1 tim

○ 2-5 tim

○ 6-10 tim

○ 11-20 tim

○ Mer än 21 tim

○ Vet inte

○ Inte relevant

○ Vill inte ange

36. I vilken omfattning kan du/din närstående med ett sällsynt hälsotillstånd arbeta, gå till förskola, skola eller annan sysselsättning?

- [ ] 100%
- [ ] 75%
- [ ] 50%
- [ ] 25%
- [ ] Ingen förskola, skola eller annan sysselsättning
- [ ] Vet inte
- [ ] Vill inte ange

37. Arbetar/studerar du/din närstående i mindre utsträckning än 100% på grund av det sällsynta hälsotillståndet?

- ○ Ja, förtydliga vid behov ______
- ○ Nej
- ○ Vet inte
- ○ Vill inte ange

Etikprövningsmyndigheten
2023-06900-01-435533
2023-11-06

**38. Vilken sysselsättning har du/din närstående?
Skola omfattar alla stadier i grundskola, gymnasium och högskola/universitet**

- ○ Förskola
- ○ Skola
- ○ Annan utbildning
- ○ Arbete
- ○ Annan sysselsättning
- ○ Ingen sysselsättning
- ○ Vet inte
- ○ Vill inte ange

39. Upplever du/din närstående att arbetsgivaren/skolpersonal (eller liknande) har kunskap om det sällsynta hälsotillståndet, symtomen och hur arbetslivet, skolvardagen eller liknande påverkas?

- ◯ Ja
- ◯ Nej. Vad saknar du ? [ ]
- ◯ Vet inte
- ◯ Vill inte ange

40. Har du/din närstående mötts av förståelse på arbetsplatsen/skolan (eller liknande) för de utmaningar du/hen har utifrån din/sin diagnos?

- ◯ Ja
- ◯ Nej. Vad saknar du? [ ]
- ◯ Vet inte
- ◯ Vill inte ange

Etikprövningsmyndigheten
2023-06900-01-435533
2023-11-06

41. Har du/din närstående fått det stöd och de anpassningar på arbetsplatsen/skolan (eller liknande) som du/hen eventuellt har behov av?

- ◯ Ja
- ◯ Nej. Vad saknar du? [ ]
- ◯ Vet inte
- ◯ Vill inte ange

Frågorna nedan handlar om din/din närståendes kontakt med viktiga samhällsfunktioner.

42. Är du/din närstående i kontakt med andra samhällsfunktioner som du/hen upplever saknar kunskap om det sällsynta hälsotillståndet och som inte erbjuder det stöd och de anpassningar du/din närstående eventuellt har behov av?

- ☐ Försäkringskassan

- [ ] Arbetsförmedlingen
- [ ] Annan, nämligen: ______
- [ ] Nej, jag har ingen kontakt med andra samhällsfunktioner
- [ ] Jag har kontakt med andra samhällsfunktioner men upplever att det fungerar bra
- [ ] Vet inte
- [ ] Vill inte ange

43. Upplever du/din närstående att samhällsfunktioner utanför vården har förändrat sitt bemötande gentemot dig/din närstående över tid, om du/hen tittar tillbaka tio år?

- ○ Ja, bemötandet har förbättrats (ge gärna exempel på förbättring) ______
- ○ Ja, bemötandet har försämrats (ge gärna exempel på försämring) ______
- ○ Nej
- ○ Vet inte
- ○ Inte relevant
- ○ Vill inte ange

Etikprövningsmyndigheten
2023-06900-01-435533
2023-11-06

44. Tycker du att det stöd som ges till syskon är tillräckligt?

- ○ Ja
- ○ Nej. (Vad tycker du skulle behövas?) ______
- ○ Vet inte
- ○ Vill inte ange
- ○ Inte relevant

Frågorna nedan kommer handla om din/din närståendes åldrande.

45. Anser du/din närstående att det finns tillräcklig kunskap om hur livet

med det sällsynta hälsotillståndet kommer se ut när du/din närstående blir äldre?

	Ja	Nej	Vet inte	Vill inte ange	Ej relevant
Hos dig/din närstående	○	○	○	○	○
Hos dina/dina närståendes vårdkontakter	○	○	○	○	○
Hos de samhällsfunktioner utanför vården som du/din närstående är i kontakt med?	○	○	○	○	○

46. Tycker du att det finns tillräckligt med hjälp och stöd från vård och samhällsfunktioner när du/din närstående blir äldre?

- ○ Ja
- ○ Nej. (Vad saknar du?) ____________
- ○ Vet inte
- ○ Vill inte ange
- ○ Inte relevant

Etikprövningsmyndigheten
2023-06900-01-435533
2023-11-06

Frågorna nedan handlar om hälso- och sjukvård.

47. Har du/din närstående någon gång under de senaste 6 månaderna besökt hälso- och sjukvården?

	Nej	Ja, 1 gång	Ja, 2-3 gånger	Ja, fler än 3 gånger	Vet inte	Inte relevant
Primärvård fysiskt	○	○	○	○	○	○
Primärvård digitalt (kry, doktor24, …)	○	○	○	○	○	○
Specialistvård	○	○	○	○	○	○
Sjukhus	○	○	○	○	○	○
Habilitering	○	○	○	○	○	○
Rehabilitering	○	○	○	○	○	○

	Nej	Ja, 1 gång	Ja, 2-3 gånger	Ja, fler än 3 gånger	Vet inte	Inte relevant
Annat (specificera nedan) [ ]	○	○	○	○	○	○

48. Hur stor del av din/din närståendes vård bedömer du sker i primärvården? Dvs. på en vårdcentral/hälsocentral.

- ○ Mer än hälften av vården
- ○ Mindre än hälften av vården
- ○ Blandat
- ○ Vet inte, tveksam
- ○ Vill inte ange
- ○ Inte relevant

Etikprövningsmyndigheten
2023-06900-01-435533
2023-11-06

49. Ange hur stort förtroende du/din närstående har för:

	Mycket stort	Ganska stort	Varken eller	Ganska litet	Mycket litet	Inte alls	Vet inte	Inte relevant
Hälso- och sjukvården	○	○	○	○	○	○	○	○
Personalen inom hälso- och sjukvården	○	○	○	○	○	○	○	○
Sjukhusen	○	○	○	○	○	○	○	○
Personalen på sjukhusen	○	○	○	○	○	○	○	○
Hälso-/vårdcentraler	○	○	○	○	○	○	○	○
Personalen hos hälso-/vårdcentraler	○	○	○	○	○	○	○	○

50. Upplever du/din närstående att kunskapsnivån inom vården är tillräcklig för att ge rätt behandling/uppföljning av det sällsynta hälsotillståndet? (Tänk på de senaste två åren)

- Ja
- Nej
- Vet inte
- Vill inte ange
- Inte relevant

51. Ange hur väl du/din närstående instämmer med nedan påstående:

	Instämmer helt	Instämmer delvis	Varken eller	Tar delvis avstånd	Tar helt avstånd	Vet inte	Vill inte ange	Inte relevant
Jag/min närstående har tillgång till den hälso- och sjukvård jag/hen behöver.	○	○	○	○	○	○	○	○
Upplever du/din närstående att du/hen någon gång blivit nekad vård pga ditt/sitt sällsynta hälsotillstånd? (tex inte blivit mottagen av en akutmottagning, inte fått behandling för halsont eller annan vanlig åkomma)	○	○	○	○	○	○	○	○

Etikprövningsmyndigheten
2023-06900-01-435533
2023-11-06

52. I vilken utsträckning upplever du/din närstående att:

	I mycket hög grad	I hög grad	I låg grad	Inte alls	Vet inte, tveksam	Vill inte ange	Inte relevant
Vården uppmuntrar till prevention/förebyggande av god hälsa?	○	○	○	○	○	○	○

53. Har du/din närstående fått en individuell vårdplan, där mål och tidsplan för behandlingar beskrivs?

- Ja, skriftlig
- Ja, muntlig

- ◯ Nej, varken skriftlig eller muntlig
- ◯ Vet inte vad en individuell vårdplan är
- ◯ Vet inte
- ◯ Vill inte ange
- ◯ Inte relevant

54. Har du/din närstående tillgång till habilitering?

- ◯ Ja
- ◯ Nej jag/hen behöver inte
- ◯ Nej, men jag/hen anser att jag/hen har behov av det
- ◯ Vet inte, tveksam
- ◯ Vill inte ange
- ◯ Inte relevant

Etikprövningsmyndigheten
2023-06900-01-435533
2023-11-06

55. Saknar du/din närstående information om ditt/din närståendes sällsynta hälsotillstånd?

- ◯ Ja. (Ge gärna exempel på vilken information du/hen saknar) [ ]
- ◯ Nej
- ◯ Vet inte
- ◯ Vill inte ange
- ◯ Inte relevant

Frågorna nedan handlar om din/dina närståendes vårdmöten

Vi ber dig besvara i vilken grad du instämmer i följande påståenden utifrån två tidsperspektiv. **Först** utgår du från hur du **generellt** upplever dina kontakter i vården. **Sedan** utgår du från hur du **upplevt vården** under covid-19.

56. I vilken utsträckning upplever du att följande uttalanden stämmer för dig

som lever med ett sällsynt hälsotillstånd eller din närstående generellt:

	I mycket hög grad	I hög grad	I låg grad	Inte alls	Tveksam, vet ej	Inte relevant
Att det är lätt att få träffa vårdpersonal när du har behövt det	○	○	○	○	○	○
Att samordningen av dina kontakter med vården sker på ett ändamålsenligt sätt	○	○	○	○	○	○
Att du känner dig delaktig i besluten om hur din vård utformas och genomförs	○	○	○	○	○	○
Att vården är anpassad till dina behov och önskemål	○	○	○	○	○	○
Att vården hjälper dig att själv hantera/förbättra din egen hälsa, t.ex. genom stöd till egenvård	○	○	○	○	○	○

Etikprövningsmyndigheten
2023-06900-01-435533
2023-11-06

57. I vilken utsträckning instämmer du med samma uttalanden vad gäller de senaste 12 månaderna, under Covid-19

	I mycket hög grad	I hög grad	I låg grad	Inte alls	Tveksam, vet ej	Inte relevant
Att det är lätt att få träffa vårdpersonal när du har behövt det	○	○	○	○	○	○
Att samordningen av dina kontakter med vården sker på ett ändamålsenligt sätt	○	○	○	○	○	○
Att du känner dig delaktig i besluten om hur din vård utformas och genomförs	○	○	○	○	○	○
Att vården är anpassad till dina behov och önskemål	○	○	○	○	○	○
Att vården hjälper dig att själv hantera/förbättra din egen hälsa, t.ex. genom stöd till egenvård	○	○	○	○	○	○

58. Upplever du att vårdens bemötande har förändrats över tid, om du tittar tillbaka tio år?

- Ja, bemötandet har förbättrats. (Ge gärna exempel och ange tidsram i år)
- Ja, bemötandet har försämrats. (Ge gärna exempel och ange tidsram i år)
- Nej
- Vet inte
- Vill inte ange
- Inte relevant

Det pågår ett utvecklingsarbete för att stärka primärvården. För att bidra med kunskap i detta arbete vill Myndigheten för vård- och omsorgsanalys veta mer om vad medlemmarna i Riksförbundet Sällsynta diagnoser tycker är viktigt. Dina fritextsvar i frågan nedan kommer oavkortat förmedlas till Myndigheten för vård- och omsorgsanalys.

59. Om du utgår från dina erfarenheter, finns det något du tycker har fungerat särskilt bra med primärvården (t.ex. vårdcentraler/hälsocentraler)?

500 tecken kvar

60. Om du utgår från dina erfarenheter, finns det något du anser borde utvecklas för att primärvården (t.ex. vårdcentraler/hälsocentraler) bättre ska möta dina behov?

500 tecken kvar

61. Skulle du och din familj vara hjälpt av att ha en kontaktperson som hjälper dig med samordning och kontinuitet i dina kontakter inom vården

Etikprövningsmyndigheten
2023-06900-01-435533
2023-11-06

och övriga samhällsfunktioner?

- ◯ Ja
- ◯ Nej
- ◯ Vet inte
- ◯ Vill inte ange
- ◯ Inte relevant

62. Har du/din närstående en eller flera fasta vårdkontakter?

- ◯ Ja, en
- ◯ Ja, flera
- ◯ Nej, men skulle ha behov av
- ◯ Nej och jag behöver inte
- ◯ Jag är tveksam om jag har en/flera
- ◯ Jag vet inte vad en fast vårdkontakt är
- ◯ Vill inte ange
- ◯ Inte relevant

Etikprövningsmyndigheten
2023-06900-01-435533
2023-11-06

Frågor nedan rör din/din närståendes fasta vårdkontakter

63. Vilken yrkeskategori har dina/din närståendes fasta vårdkontakt/er? Du kan välja flera svarsalternativ

- ☐ Läkare inom primärvård
- ☐ Läkare inom specialistvård/sjukhus
- ☐ Sjuksköterska inom primärvård
- ☐ Sjuksköterska inom specialistvård/sjukhus
- ☐ Psykolog
- ☐ Kurator
- ☐ Fysioterapeut/sjukgymnast

- [ ] Vet inte
- [ ] Vill inte ange
- [ ] Inte relevant
- [ ] Annan

64. Upplever du att din/din närståendes fasta vårdkontakt/er hjälper dig i kontakterna med vården samt med att samordna vårdens insatser?

- ( ) Ja. Förtydliga hur
- ( ) Nej. Varför inte
- ( ) Vet inte
- ( ) Vill inte ange
- ( ) Inte relevant

Etikprövningsmyndigheten
2023-06900-01-435533
2023-11-06

Frågorna nedan handlar om expertkunskapen kring det sällsynta hälsotillståndet.

65. Var anser du/din närstående att den bästa informationen om det sällsynta hälsotillståndet finns? Du kan välja max tre informationskällor.

- [ ] Läkare
- [ ] Annan vårdpersonal
- [ ] Centrum för Sällsynta diagnoser vid de sex universitetssjukhusen
- [ ] Expertteam vid de sex universitetssjukhusen
- [ ] Socialstyrelsens kunskapsdatabas för sällsynta hälsotillstånd
- [ ] Orpha.net, den europeiska databasen för sällsynta hälsotillstånd
- [ ] Riksförbundet Sällsynta diagnoser
- [ ] Andra diagnosförbund, -föreningar eller -nätverk
- [ ] Sociala medier bloggar
- [ ] Sociala medier Facebook/grupper
- [ ] På nätet (googlar)

- [ ] Vänner bekanta
- [ ] Annat

66. Har du/din närstående varit i kontakt med/fått behandling av någon som är expert på det sällsynta hälsotillståndet?

- ○ Ja
- ○ Nej
- ○ Vet inte
- ○ Vill inte ange
- ○ Inte relevant

Etikprövningsmyndigheten
2023-06900-01-435533
2023-11-06

67. Har du/ din närstående varit i kontakt med/fått behandling av ett medicinskt expertcenter eller expertteam för ditt/din närståendes sällsynta hälsotillstånd?

- ○ Ja
- ○ Nej
- ○ Vet inte
- ○ Vet inte vad ett medicinskt expertcenter eller expertteam är
- ○ Vill inte ange
- ○ Inte relevant

Frågorna nedan handlar om läkemedel och behandling för det sällsynta hälsotillståndet.

68. Tycker du/din närstående att processen för att utvärdera och fatta beslut om tillgång till läkemedel mot sällsynta sjukdomar i Sverige är:

	Ja	Nej	Vet inte hur processen fungerar	Vill inte ange	Inte relevant
Tydlig	○	○	○	○	○
Öppen	○	○	○	○	○

	Ja	Nej	Vet inte hur processen fungerar	Vill inte ange	Inte relevant
Lätt att förstå	◯	◯	◯	◯	◯

69. Finns det läkemedelsbehandling för det sällsynta hälsotillståndet som du/din närstående har?

◯ Nej det finns ingen läkemedelsbehandling för det sällsynta hälsotillståndet

◯ Ja det finns en läkemedelsbehandling som är tillgänglig i Sverige

◯ Ja, det finns en läkemedelsbehandling som är godkänd i Europa men som inte är tillgänglig i Sverige

◯ Vet inte

◯ Vill inte ange

◯ Inte relevant

Etikprövningsmyndigheten
2023-06900-01-435533
2023-11-06

70. Behandlas du/din närstående med något av de läkemedel som finns för det sällsynta hälsotillståndet?

◯ Ja, behandlingen genomförs i Sverige med läkemedelsförmån

◯ Ja, behandlingen genomförs i Sverige på egen bekostnad

◯ Ja, behandlingen genomförs utanför Sverige i enlighet med EU:s patienträrlighetsdirekiv. Ange gärna var [ ]

◯ Ja, behandligen genomförs utanför Sverige på egen bekostnad. Ange gärna var [ ]

◯ Nej ingen behandling genomförs

◯ Vet inte

◯ Vill inte ange

◯ Inte relevant

71. Vad är anledningen till att du/din närstående inte behandlas med något av de läkemedel som finns för det sällsynta hälsotillståndet?

- Jag/min närstående önskar inte behandlas med de läkemedel som finns för det sällsynta hälsotillståndet
- Jag/min närstående väntar på besked om behandling med de läkemedel som finns för det sällsynta hälsotillståndet
- Jag/min närstående har fått besked om att hälso- och sjukvården inte kommer att erbjuda behandling med de läkemedel som finns för det sällsynta hälsotillståndet.
- Vet inte
- Vill inte ange
- Inte relevant
- Annat

Frågorna nedan handlar om kliniska läkemedelsprövningar.

72. Deltar du/din närstående i en klinisk läkemedelsprövning?

Etikprövningsmyndigheten
2023-06900-01-435533
2023-11-06

- Ja
- Nej
- Vet inte
- Vet inte vad en klinisk läkemedelsprövning är
- Vill inte ange

73. Har du/din närstående tidigare deltagit i en klinisk läkemedelsprövning?

- Ja
- Nej
- Vet inte
- Vill inte ange

74. Skulle du/din närstående vilja delta i en klinisk läkemedelsprövning?

- Ja

- ○ Nej
- ○ Vet inte
- ○ Vill inte ange

75. Innan vi går vidare till de två sista delarna som handlar om munhälsa och covid-19, finns det något område som berör det sällsynta hälsotillståndet som du/din närstående vill lyfta?

- ○ Nej
- ○ Ja, nämligen ______

Frågorna nedan handlar om mun- och tandhälsa.

76. Har du/din närstående regelbunden kontakt med tandvården? (regelbunden = minst en gång om året)

Etikprövningsmyndigheten
2023-06900-01-435533
2023-11-06

- ○ Ja
- ○ Nej
- ○ Vet inte
- ○ Vill inte ange

77. Har du/din närstående någonsin avstått från tandvård på grund av något av följande? Du kan välja flera svarsalternativ.

- ☐ Lång resa
- ☐ Tidsbrist
- ☐ För dyrt
- ☐ Nej
- ☐ Vet inte
- ☐ Vill inte ange
- ☐ Inte relevant

☐ Annat

78. Känner du/din närstående dig delaktig i planeringen av den egna tandvårdsbehandlingen?

- ◯ Ja
- ◯ Nej, jag känner inte behov av det
- ◯ Nej, men skulle vilja vara mer delaktig. Förtydliga gärna
- ◯ Vet inte
- ◯ Vill inte ange
- ◯ Inte relevant

Etikprövningsmyndigheten
2023-06900-01-435533
2023-11-06

79. Har det varit svårigheter att byta från barntandvård till vuxentandvård?

- ◯ Ja. Förtydliga gärna
- ◯ Nej
- ◯ Vet inte
- ◯ Vill inte ange
- ◯ Inte relevant

80. Vad fungerar bra med tandvården?

500 tecken kvar

81. Vad fungerar mindre bra med tandvården?

500 tecken kvar

82. Kan symptom från tänder och/eller munnen ingå som en del i ditt/din närståendes sällsynta hälsotillstånd?

- ◯ Ja
- ◯ Nej
- ◯ Vet inte
- ◯ Vill inte ange
- ◯ Inte relevant

Etikprövningsmyndigheten
2023-06900-01-435533
2023-11-06

Frågor nedan handlar om hur det sällsynta hälsotillståndet påverkar din tand- och munhälsa.

83.

	Ja	Nej	Vet inte	Vill inte ange	Inte relevant
Besöker du/din närstående tandläkare oftare än en gång per år p.g.a det sällsynta hälsotillståndet?	◯	◯	◯	◯	◯
Har du/din närstående fått tydlig information från din tandläkare hur den sällsynta diagnosen eller ev. medicinering kan påverka din tandhälsa?	◯	◯	◯	◯	◯
Har du /din närstående fått information hur du kan förebygga och underhålla god munhygien i samband med din diagnos?	◯	◯	◯	◯	◯

84. Har du/din närstående tillgång till hjälpmedel gällande tand- och munhälsa?

- ◯ Ja

◯ Nej, jag behöver inga

◯ Nej, men jag skulle behöva till exempel följande

◯ Vet inte

◯ Vill inte ange

◯ Inte relevant

85. Känner din/din närståendes tandläkare till det sällsynta hälsotillståndet?

◯ Ja

◯ Nej

◯ Vet inte

◯ Har ingen (fast) tandläkare

◯ Vill inte ange

◯ Inte relevant

Etikprövningsmyndigheten
2023-06900-01-435533
2023-11-06

86. Har du/din närstående varit hos, eller har erbjudits tid för, en specialisttandläkarbedömning med anledning av det sällsynta hälsotillståndet?

◯ Ja

◯ Nej och jag behöver ingen

◯ Nej, men jag anser att jag skulle behöva det

◯ Vet inte

◯ Vill inte ange

◯ Inte relevant

87. Hur fick du/din närstående kontakt med specialisttandvården?

◯ Remiss från sjukvården

- Remiss från ordinarie tandläkare
- Sökte kontakt själv
- Vet inte
- Vill inte ange
- Inte relevant
- Annat

88. Är du/din närstående 24 år eller äldre?

- Ja, 24 år eller äldre
- Nej, yngre än 24 år

Etikprövningsmyndigheten
2023-06900-01-435533
2023-11-06

89. Har du/din närstående behövt genomföra större behandling för dina/sina tänder utöver undersökningar och enklare förebyggande behandling som t ex putsa tänderna, ta bort lite tandsten, fluorlackning?

- Ja
- Nej
- Vet inte
- Vill inte ange
- Inte relevant

90. Har du/din närstående fått information om olika tandvårdsstöd som eventuellt kan vara aktuella för dig/hen?

- Ja
- Nej
- Vet inte
- Vill inte ange
- Inte relevant

91. Vem informerade om tandvårdsstöden?

- Tandläkare
- Tandsköterska
- Tandhygienist
- Läkare
- Närstående
- Patientförening
- Sökt information själv
- Vet ej
- Vill inte ange
- Inte relevant
- Annan

Etikprövningsmyndigheten
2023-06900-01-435533
2023-11-06

92. Har du/din närstående fått ekonomiskt bidrag/tandvårdsstöd för tandbehandling utöver det vanliga högkostnadsskyddet?

T ex som Led i sjukdom, F11 sällsynt diagnos med eller utan orofacial funktionsnedsättning, Särskilt Tandvårdsbidrag, Privat försäkring eller annan ersättning?

- Ja
- Nej. Förtydliga vid behov
- Sökt men fått avslag. Förtydliga vid behov
- Vet inte
- Vill inte ange
- Inte relevant

93. Har det varit svårt att få läkarintyg för att söka tandvårdsstöd?

- ○ Ja, förtydliga vid behov [ ]
- ○ Nej, förtydliga vid behov [ ]
- ○ Vet inte
- ○ Vill inte ange
- ○ Inte relevant

Nedan följer frågor kring tandvården till dig/din närstående som är yngre än 24 år.

94. Anser du/din anhörig att du behöver/behövde tandställning?

- ○ Ja
- ○ Nej
- ○ Vet inte
- ○ Vill inte ange
- ○ Inte relevant

Etikprövningsmyndigheten
2023-06900-01-435533
2023-11-06

95. Har du/din anhörig erbjudits bedömning av tandregleringsspecialist?

- ○ Ja
- ○ Ja, men tackat nej. Förtydliga vid behov [ ]
- ○ Nej, men jag hade velat ha en. Förtydliga vid behov [ ]
- ○ Nej och jag har inte behövt
- ○ Vet inte
- ○ Vill inte ange
- ○ Inte relevant

96. Anser tandvården att du/din anhörig behöver/behövde tandställning?

- ○ Ja
- ○ Ja, men jag anser inte det. Förtydliga vid behov [ ]

◯ Nej, men jag anser det. Förtydliga vid behov

◯ Nej, och jag instämmer

◯ Vet inte

◯ Vill inte ange

◯ Inte relevant

97. Har du/din anhörig erbjudits tandställning?

◯ Ja

◯ Ja, men jag har tackat nej. Förtydliga vid behov

◯ Nej, men jag hade velat ha. Förtydliga vid behov

◯ Nej, och jag instämmer

◯ Vet inte

◯ Vill inte ange

◯ Inte relevant

Etikprövningsmyndigheten
2023-06900-01-435533
2023-11-06

Frågor nedan handlar om covid-19 och dess påverkan på din/din närståendes hälsa och sjukvård

98. Hur har tillgången till vård fungerat under utbrottet av covid-19 (februari 2020 fram tills nu)?

◯ Som tidigare, utan störning

◯ Med vissa störningar

◯ Med betydande störningar

◯ Har inte fungerat alls

◯ Vet inte

◯ Vill inte ange

◯ Inte relevant

99. Vilka störningar avseende tillgång till vården har du/din närstående upplevt?

500 tecken kvar

100. Har annan vård eller stöd ställts in, bortsett från medicinsk vård?

- Ja, specificera i gärna vilken typ av vård eller stöd (exempelvis socialt stöd via Socialtjänstlagen eller LSS, re-/habilitering, fysioterapi, logoped, besök hos kurator/psykolog eller liknande).
- Nej
- Inte relevant

Etikprövningsmyndigheten
2023-06900-01-435533
2023-11-06

101. Har du/din närstående redan vaccinerats för covid-19?

- Ja
- Nej
- Vet ej

102. Har du/din närstående fått biverkningar av covid-19-vaccinet?

- Nej
- Ja, lätta biverkningar. Vilka då:
- Ja, svåra biverkningar. Vilka då:

103. Kommer du/din närstående vaccineras för covid-19?

- Ja
- Nej
- Vet ej
- Vill inte ange

104. Om du/din närstående är osäker eller har bestämt dig/sig att inte vaccineras för covid-19, ange anledningen nedan

- På grund av det sällsynta hälsotillståndet
- På grund av annan hälsorelaterad orsak
- Vet inte
- Vill inte ange
- Av andra skäl

Etikprövningsmyndigheten
2023-06900-01-435533
2023-11-06

105. Om du/din närstående inte kommer att vaccineras för covid-19, ange vems rekommendation du/hen följer.

- [ ] Rekommendation från svensk läkare/sjukvården
- [ ] Rekommendation från internationella läkare/sjukvården
- [ ] Rekommendation från annan aktör i Sverige till exempel myndigheter, andra samhällsaktörer, andra aktörer som t.ex. diagnosföreningar eller annat. Ange gärna vilken/vilka
- [ ] Rekommendation från annan aktör utanför Sverige till exempel myndigheter, samhällsaktörer, andra aktörer som t.ex.diagnosföreningar med mera. Ange gärna vilken/vilka
- [ ] Utan rekommendation från någon särskild
- [ ] Annans, nämligen
- [ ] Vet inte
- [ ] Vill inte ange
- [ ] Inte relevant
