# Supplementary material for: Diagnostic delay, care needs, and trust in healthcare among persons with rare disease and their next of kin living in Sweden
Source: J Community Genet. 2026 Aug 20;17(5):102. doi: 10.1007/s12687-026-00931-6 (PMC13493750; doi:10.1007/s12687-026-00931-6)
Supplement: Supplementary file 3 [file 12687_2026_931_MOESM3_ESM.docx]

Diagnostic delay, Care needs, and Trust in Healthcare among persons with Rare Disease and their next of kin living in Sweden

**Maria Johansson Soller**^1,5,^ Jamie Linnea Luckhaus^3^, Stephanie Juran^4^, **Charlotta Ingvoldstad Malmgren**^1, 2, 6^

^1,^ Karolinska Institutet, Department of Molecular Medicine and Surgery, Stockholm, Sweden

^2^ Uppsala University, Center for research ethics and bioethics, Uppsala, Sweden

^3^ Uppsala University, Participatory eHealth and Health Data, Department of Women’s and Children’s Health, Uppsala, Sweden

^4^ Rare diseases Sweden, Swedish umbrella organization for patients and relatives, Sundbyberg, Sweden

^5^ Akademiska University Hospital, Department of Clinical Genetics, Uppsala, Sweden

^6^Center for Rare Diseases, Clinical genetics, Karolinska University hospital

* Corresponding author:

Charlotta Ingvoldstad Malmgren

[Charlotta.ingvoldstad-malmgren@regionstockholm.se](mailto:Charlotta.ingvoldstad-malmgren@regionstockholm.se)

ORCID ID: 0000-0003-0754-5065

# Appendix

## Appendix I: STROBE checklist

## Appendix II: Original survey questions (Swedish)

### Appendix III: Supplementary Material

**Table 1**. Socio-demographic characteristics.

|  |  | Count | % |
| --- | --- | --- | --- |
| **Respondent type** (*n* = 940) |  |  |  |
| Patient answering for self |  | 410 | 43.6 |
| Patient and caregiver, answering for self |  | 107 | 11.4 |
| Caregiver answering for next of kin/loved one |  | 408 | 43.4 |
| Patient and caregiver answering for next of kin/loved one |  | 9 | 1 |
| **Gender** (*n =* 942) |  |  |  |
| Woman |  | 554 | 58.8 |
| Man |  | 367 | 39 |
| Other ^1^ |  | 6 | 0.6 |
| Don’t want to specify / Missing |  | 15 | 1.5 |
| **Age** (*n* = 940) |  |  |  |
| 6 years or younger |  | 46 | 5 |
| 7 – 11 years |  | 77 | 8.2 |
| 12 – 18 years |  | 79 | 8.4 |
| 19 – 24 years |  | 58 | 6.2 |
| 25 – 34 years |  | 115 | 12.2 |
| 35 – 44 years |  | 106 | 11.3 |
| 45 – 54 years |  | 126 | 13.4 |
| 55 – 64 years |  | 149 | 15.9 |
| 65 – 74 years |  | 114 | 12.1 |
| 75 years or older |  | 50 | 5.3 |
| **Country of birth** (*n* = 936) |  |  |  |
| Sweden |  | 899 | 96.1 |
| Northern Europe (not Sweden) |  | 12 | 1.3 |
| Europe (not Northern) |  | 14 | 1.5 |
| Global (not Europe) |  | 11 | 1.2 |
| **Highest level of education** (*n* = 937) |  |  |  |
| Currently in pre-/primary school |  | 107 | 11.4 |
| Primary school |  | 69 | 7.4 |
| Special education, Primary school |  | 45 | 4.8 |
| Secondary education/High school |  | 228 | 24.3 |
| Special education, Secondary/High school |  | 83 | 8.9 |
| Higher education/University/College |  | 322 | 34.4 |
| Other |  | 76 | 7.6 |
| **Employment status** (*n* = 779) |  |  |  |
| Employed |  | 301 | 38.7 |
| Unemployed |  | 116 | 15 |
| In pre-school |  | 31 | 4 |
| In school |  | 195 | 25 |
| Other education |  | 15 | 1.9 |
| *Don’t know* or *Prefer not to specify* |  | 18 | 2.3 |
